# Disruption of ER ion homeostasis maintained by an ER anion channel CLCC1 contributes to ALS-like pathologies

**DOI:** 10.1038/s41422-023-00798-z

**Published:** 2023-05-04

**Authors:** Liang Guo, Qionglei Mao, Ji He, Xiaoling Liu, Xuejiao Piao, Li Luo, Xiaoxu Hao, Hanzhi Yu, Qiang Song, Bailong Xiao, Dongsheng Fan, Zhaobing Gao, Yichang Jia

**Affiliations:** 1 Tsinghua-Peking Joint Center for Life Sciences, Tsinghua University, Beijing, China; 2 School of Life Sciences, Tsinghua University, Beijing, China; 3 School of Medicine, Tsinghua University, Beijing, China; 4 IDG/McGovern Institute for Brain Research, Tsinghua University, Beijing, China; 5 CAS Key Laboratory of Receptor Research, State Key Laboratory of Drug Research, Shanghai Institute of Materia and Medica, Chinese Academy of Sciences, Shanghai, China; 6 University of Chinese Academy of Sciences, Beijing, China; 7 Department of Neurology, Peking University Third Hospital, Beijing, China; 8 School of Pharmaceutical Sciences, Tsinghua University, Beijing, China; 9 Tsinghua Laboratory of Brain and Intelligence, Beijing, China; 10 School of Pharmaceutical Sciences, Zhejiang University, Hangzhou, Zhejiang China; 11 School of Medicine, Zhejiang University City College, Hangzhou, Zhejiang China; 12 Beijing Municipal Key Laboratory of Biomarker and Translational Research in Neurodegenerative Diseases, Beijing, China

**Keywords:** Mechanisms of disease, Endoplasmic reticulum

## Abstract

Although anion channel activities have been demonstrated in sarcoplasmic reticulum/endoplasmic reticulum (SR/ER), their molecular identities and functions remain unclear. Here, we link rare variants of *Chloride Channel CLIC Like 1* (*CLCC1*) to amyotrophic lateral sclerosis (ALS)-like pathologies. We demonstrate that CLCC1 is a pore-forming component of an ER anion channel and that ALS-associated mutations impair channel conductance. CLCC1 forms homomultimers and its channel activity is inhibited by luminal Ca^2+^ but facilitated by phosphatidylinositol 4,5-bisphosphate (PIP2). We identified conserved residues D25 and D181 in CLCC1 N-terminus responsible for Ca^2+^ binding and luminal Ca^2+^-mediated inhibition on channel open probability and K298 in CLCC1 intraluminal loop as the critical PIP2-sensing residue. CLCC1 maintains steady-state [Cl^–^]_ER_ and [K^+^]_ER_ and ER morphology and regulates ER Ca^2+^ homeostasis, including internal Ca^2+^ release and steady-state [Ca^2+^]_ER_. ALS-associated mutant forms of CLCC1 increase steady-state [Cl^–^]_ER_ and impair ER Ca^2+^ homeostasis, and animals with the ALS-associated mutations are sensitized to stress challenge-induced protein misfolding. Phenotypic comparisons of multiple *Clcc1* loss-of-function alleles, including ALS-associated mutations, reveal a CLCC1 dosage dependence in the severity of disease phenotypes in vivo. Similar to *CLCC1* rare variations dominant in ALS, 10% of K298A heterozygous mice developed ALS-like symptoms, pointing to a mechanism of channelopathy dominant-negatively induced by a loss-of-function mutation. Conditional knockout of *Clcc1* cell-autonomously causes motor neuron loss and ER stress, misfolded protein accumulation, and characteristic ALS pathologies in the spinal cord. Thus, our findings support that disruption of ER ion homeostasis maintained by CLCC1 contributes to ALS-like pathologies.

## Introduction

Although Cl^–^ is the most abundant anion in living cells, chloride currents and their functional significance had been understudied until the CLC family of chloride channels and cystic fibrosis transmembrane conductance regulator (CFTR) were cloned and their dysfunctions were linked to human diseases.^1–4^ In addition to those on the cell surface, Cl^–^ channels have long been proposed to exist in the intracellular membrane-bound organelles.^3,5^ However, the previously postulated intracellular Cl^–^ channels, like chloride channel Ca^2+^-activated (CLCAs) and chloride intracellular channels (CLICs), are now considered unlikely to function as anion channels.^6,7^ Therefore, the molecular identities and functions of organellar anion channels, including those in the sarcoplasmic reticulum/endoplasmic reticulum (SR/ER), remain largely unknown.

As the major internal Ca^2+^ store, Ca^2+^ release from SR/ER is mediated by two cation channels, ryanodine receptors (RyRs) and inositol 1,4,5-trisphosphate receptors (IP3Rs).^8–10^ During the release, SR/ER membrane is charged upon the Ca^2+^ efflux, which hinders the continued Ca^2+^ release. Previously reported TRimeric Intracellular Cation channels (TRICs) act as counter-ion channels to balance the loss of positive charges from the SR/ER due to the release.^11^ In addition to cations, anions have also been proposed to function as counter-ion for the release, and various Cl^–^ channel activities have been long demonstrated in microsome preparations.^12–17^ A previous study using mouse forward genetics revealed that loss of Chloride Channel CLIC Like 1 (CLCC1), an ER-resident protein,^18,19^ leads to ER stress and neurodegeneration.^19^ However, despite the name, CLCC1 has little sequence similarity with CLIC family members or any known ion channels. In addition, the question remains whether the recorded chloride currents in the microsome prepared from the CLCC1-overexpressing cells were actually mediated by CLCC1.^20,21^ Therefore, further evidence is needed to know whether CLCC1 functions as an anion channel.

Here, we demonstrate that CLCC1 is a pore-forming component of an ER anion channel by incorporating purified CLCC1 into a lipid bilayer. Depletion of CLCC1 reduces ER Ca^2+^ release, probably through a counter-ion mechanism, but increases steady-state [Cl^–^]_ER_ and [K^+^]_ER_. We identified *CLCC1* rare variants in a Chinese amyotrophic lateral sclerosis (ALS) cohort. The disease-associated nonsynonymous mutations impair CLCC1 channel conductance and promote misfolded protein accumulation in the mutation knock-in mouse brain and spinal cord. Conditional removal of *Clcc1* in ChAT-positive motor neuron cell-autonomously leads to ubiquitin-positive inclusions and mislocalized TDP-43, a pathological hallmark of ALS, and motor neuron loss. Therefore, we argue that misregulation of ER ion homeostasis maintained by an ER anion channel underlies ER unfolded protein response (UPR) and the etiology of neurodegenerative diseases.

## Results

### CLCC1 forms homomultimer in the ER membrane

**Fig. 1:**
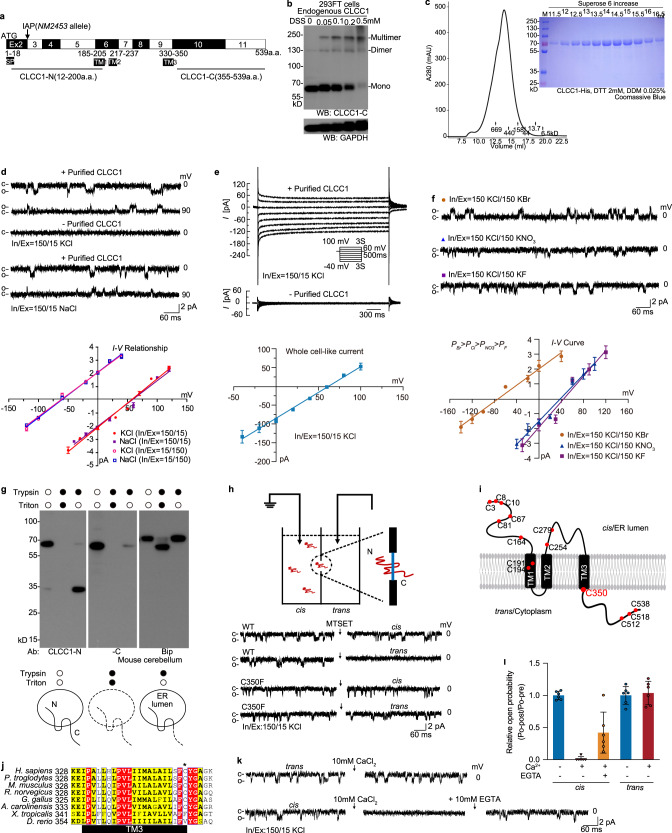

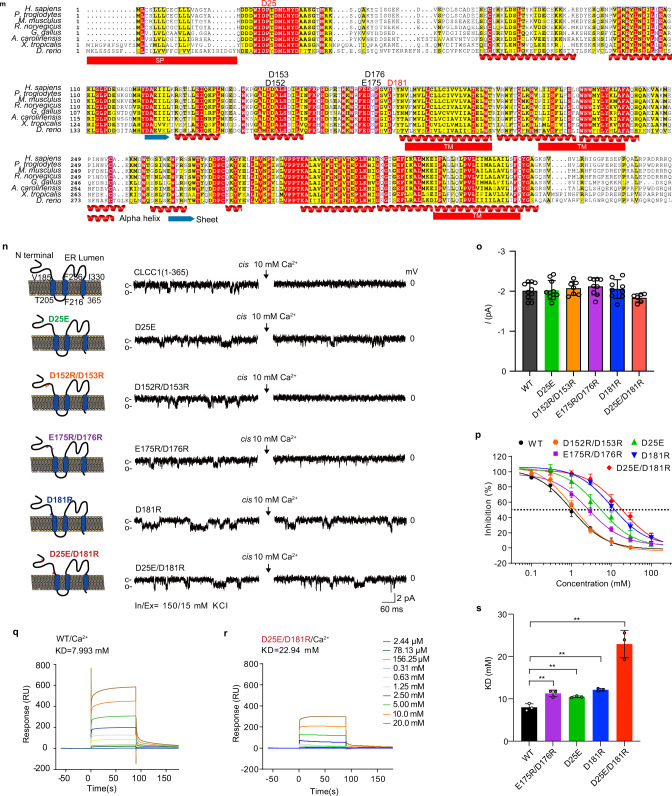
CLCC1 is a pore-forming component of an ER anion channel and luminal Ca^2+^ inhibits the channel activity. **a** Domain prediction of mouse CLCC1 (mCLCC1, NM_145543.2) that contains an SP and three TMs. The N-terminal (12–200 aa) and C-terminal polypeptides (355–539 aa) were used for generation of the N- and C-terminal antibodies, respectively. **b** Naive 293FT cells were treated with DSS at the indicated concentrations. Cell lysates were separated by SDS-PAGE and blotted with CLCC1 C-terminal antibody (CLCC1-C). **c** Chromatograph of His-tagged mCLCC1 expressed by an insect expression system and purified by Nickel column. Standard molecular weight markers are indicated. **d** Purified mCLCC1 from **c** were incorporated into planar phospholipid bilayer and single channel currents were recorded in asymmetric KCl and NaCl solutions at indicated voltages (upper). C, closed state; O, open state. Current-voltage (*I-V*) relationships in asymmetric KCl and NaCl solutions (lower). **e** Representative macroscopic currents recorded with or without the addition of purified mCLCC1 (upper) and the corresponding *I-V* curve for mCLCC1 (lower). **f** Single channel currents recorded at 0 mV with 150 mM KCl *in cis* and 150 mM KBr, 150 mM KNO_3_, or 150 mM KF *in trans* (upper). *I-V* relationships under the conditions (lower). In **d** and **e**, values are presented as mean ± SD (*n* ≥ 6). **g** Topology of CLCC1 determined by microsome preparation. Microsomal vesicles prepared from mouse cerebellum were treated with trypsin alone, or trypsin together with Triton X-100. Protein lysates were then separated by SDS-PAGE and probed with CLCC1 N- and C-terminal antibodies. As a control, Bip, an ER lumen resident, was protected from trypsinization. **h** Application of 2 mM MTSET in the *trans* but not *cis* side blocked mCLCC1 channel activity. We defined the *cis* side as the chamber we applied purified CLCC1 proteins (upper). The C350F mCLCC1 mutant was resistant to MTSET (*n* ≥ 6). **i** Cysteine residues of CLCC1, with C350 highlighted. **j** Sequence alignment of predicted TM3 of CLCC1 across different species. C350 is labeled with an asterisk. The corresponding residue of *Homo sapiens* and *Mus musculus* C350 is phenylalanine in *Xenopus*. **k** Application of 10 mM CaCl_2_ *in cis* but not *trans* reduced mCLCC1 channel activity. The inhibitory effect of Ca^2+^ was partially prevented by EGTA (10 mM). **l** Statistical analysis of normalized relative open probability (*Po*). Relative *Po*, *Po*-post/*Po*-pre (*Po* after CaCl_2_ or EGTA treatment divided by that before the treatment). Values are presented as mean ± SD (*n* = 6). **m** Sequence alignment of CLCC1 N-terminus and predicted TMs across different species. Conserved residues for potential Ca^2+^ binding were labeled. **n** Inhibition of CaCl_2_ (10 mM) on the WT and mutant CLCC1. **o** The amplitude of the WT and mutant CLCC1 current measured at 0 mV without application of CaCl_2_. **p** The dosage effect of CaCl_2_ on the channel activities. **q**–**s** The binding affinity of Ca^2+^ to the WT and mutant CLCC1 measured by SPR. The WT (**q**) or mutant (D25E/D181R, **r**) CLCC1 SPR response (resonance units, RU) to different [Ca^2+^]. *K*_D_ values of the indicated WT and mutant CLCC1 (**s**). In **o**, **p**, and **s**, values are presented as mean ± SD (*n* ≥ 6), **P < 0.01 by one-way ANOVA.

Based on its primary sequence, CLCC1 shares little sequence similarity with any known ion channels but is predicted to contain three transmembrane segments (TMs) and an N-terminal signal peptide (SP) (Fig. 1a). We generated antibodies against the N- and C-termini of CLCC1 (Supplementary information, Fig. S1a). Using the C-terminal antibody, we confirm that as suggested by a previous report,^18,19^ CLCC1 is predominantly ER-localized, as demonstrated by its co-localization with CALNEXIN, an ER-resident protein (Supplementary information, Fig. S1b). Our biochemical analysis further confirmed the ER localization of CLCC1 (Supplementary information, Fig. S1c).

To understand how CLCC1 functions in the ER, we treated human 293FT cells with disuccinimidyl suberate (DSS), a crosslinker with a spacer length of 11.4 Å. The C-terminal antibody detected high molecular weight complexes in a DSS dosage-dependent manner from whole cell lysate. From the complex sizes, we speculated that CLCC1 forms homomultimers (Fig. 1b), which was supported by co-immunoprecipitation of differentially tagged CLCC1 co-expressed in the same cells (Supplementary information, Fig. S2a) and of exogenously tagged CLCC1 with endogenous CLCC1 (Supplementary information, Fig. S2b). Consistent with our cell culture data (Fig. 1b) and a previous report,^20^ our native gel experiments suggested complex formations (~180 kD and ~360 kD) of purified full-length CLCC1 and that (~360 kD, the major band) of endogenous CLCC1 (Supplementary information, Fig. S2c). In addition, the purified full-length mouse CLCC1 (mCLCC1) gave rise to a major high molecular weight peak by chromatographic column separation (Fig. 1c). Taken together, our data suggest that CLCC1 forms homomultimers in the ER membrane.

### CLCC1 is a pore-forming component of an anion channel

Incorporation of the purified full-length mCLCC1 (Fig. 1c) into planar lipid bilayer resulted in frequent inward currents at 0 mV (–2.2 ± 0.3 pA) in asymmetric KCl solutions (In/Ex, 150/15 mM) and the currents became outward at 90 mV (1.6 ± 0.2 pA) (Fig. 1d). As a negative control, the protein purification buffer without protein gave rise to no current (Fig. 1d). Based on the fit of the current-voltage relationship, the reversal potentials were determined to be 56.8 mV (In/Ex, 150/15 mM KCl) and –60.3 mV (In/Ex, 15/150 mM KCl), which are close to the calculated values (59.2 mV and –59.2 mv, respectively) for Cl^–^ by Nernst equation, and the slope conductance was 39.9 ± 2.3 pS (mean ± SD). The permeability ratio of *P*(Cl^–^) to *P*(K^+^) is ~100–1 and similar results were obtained by using asymmetric NaCl solutions (Fig. 1d). Consistent with the single channel results, the reversal potential obtained from studying macroscopic currents was 61.9 mV in 15/150 mM KCl (In/Ex) (Fig. 1e), further supporting the anion selectivity. Both square step-like multiple-channel currents (Supplementary information, Fig. S3) and a large-scale current (80–90 pA) (Fig. 1e) suggested that CLCC1 is capable of mediating multi-channel currents.

Next, we examined CLCC1 channel permeability to various anions, including Br^–^, NO_3_^–^, and F^–^, by adding 150 mM KCl *in cis* (In) and equal electric charges of KBr, KNO_3_, or KF in the *trans* (Ex) chamber (Fig. 1f). The relative permeabilities of these anions to Cl^–^ were 17.14 (*P*_Br_/*P*_Cl_), 0.22 (*P*_NO3_/*P*_Cl_), and 0.18 (*P*_F_/*P*_Cl_), respectively, indicating an order of the CLCC1 anion selectivity of *P*_Br_ > *P*_Cl_ > *P*_NO3_ > *P*_F_, which is similar to that of CFTR.^22,23^ In these experiments, no cation permeation was detected. Collectively, our results demonstrate that CLCC1 is a pore-forming component of an anion channel.

### ER membrane topology of CLCC1 and its inhibition by luminal calcium

To examine CLCC1 topology in the ER membrane, we treated microsomes prepared from WT mouse cerebella and livers^24^ with trypsin and analyzed the remaining CLCC1 fragments with our N- and C-terminal antibodies. In the absence of Triton X-100, the N-terminus and the first and second loops of CLCC1 and an ER lumen-resident protein Bip were protected from trypsinization, but the C-terminus of CLCC1 was not (Fig. 1g; Supplementary information, Fig. S1d). As expected for membrane enclosure, the protection was disrupted by Triton X-100, suggesting that CLCC1 N-terminus and the second loop reside in ER lumen while C-terminus faces the cytoplasm.

Interestingly, when we applied methanethiosulfonate-ethyltrimethylammonium (MTSET),^25^ a membrane-impermeant thiol reagent that modifies cysteine residues, on the *trans* side but not to the *cis* side of the chamber where we applied the purified CLCC1, the CLCC1 currents were suppressed (Fig. 1h), suggesting that a specific orientation of CLCC1 in the bilayer is responsible for the current. Based on the topology (Fig. 1g), cysteine residues are located in both the cytoplasm and ER lumen sides of CLCC1 and C350 lies at the end of TM3 (Fig. 1i). Protein alignment among different species revealed that C350 is in a consecutive row of four residues (FCYG), although it is less conserved than the other three surrounding resides (Fig. 1j). Instead of FCYG in *Homo sapiens* and *Mus musculus*, FFYG appears in *Xenopus tropicalis*, which prompted us to mutate C350 to F. C350F mCLCC1 is expressed and its chromatographic behavior is similar to WT mCLCC1 (Supplementary information, Fig. S4a). Importantly, C350F restored the CLCC1 currents even when MTSET was applied on the *trans* side (Fig. 1h), suggesting that MTSET acts on C350 to modify the channel activity and the *trans* side is the CLCC1 cytoplasm side in the reconstructed lipid bilayer. Application of 4,4′-Diisothiocyano-2,2′-stilbenedisulfonic acid (DIDS), a chloride transporter/channel blocker,^4,26^ significantly inhibited CLCC1 channel activity (Supplementary information, Fig. S4b, c). Consistent with MTSET acting on C350, C350F largely restores the DIDS inhibition on channel open probability (*P*_o_).

Because ER luminal Ca^2+^ level is much higher than cytoplasmic Ca^2+^ level, we then asked whether Ca^2+^ can differentially regulate CLCC1 channel activity from ER luminal or cytoplasmic side. Applying Ca^2+^ in *cis*/ER lumen side blocked the CLCC1 channel activity, which could be partially rescued by adding equal molar EGTA, a Ca^2+^ chelating agent (Fig. 1k, l). However, the same application on *trans*/cytoplasmic side had no effect on the channel activity. Consistent with our single-channel recordings, our macroscopic recordings also supported the notion that luminal Ca^2+^ inhibits CLCC1 currents (Supplementary information, Fig. S5). Therefore, we conclude that, at least in our reconstructed lipid bilayer setting, a high concentration of Ca^2+^ at the ER lumen side inhibits CLCC1 channel activity.

### D25/D181 are the key residues responsible for Ca^2+^-dependent inhibition on the channel open probability

To identify key residues at the lumen side responsible for the Ca^2+^ inhibition, we aligned the protein sequences across different species (Fig. 1m). Several conserved negatively charged residues at N-terminus drew our attention, including D25, D152, D153, E175, D176, and D181. Because CLCC1 D25E mutation has been associated with retinitis pigmentosa,^21^ we took D25E into account. In addition, we generated D152R/D153R, E175R/D176R, D181R, and D25E/D181R mutant CLCC1 for Ca^2+^-binding affinity and channel activity measurements.

Because the C-terminus of CLCC1 resides at the cytoplasmic/*trans* side, we truncated the C-terminus (1–365) and recorded a sound current that could be inhibited by Ca^2+^ (10 mM) application at the *cis* side (Fig. 1n). In addition, all mutant CLCC1 yielded reasonable currents as WT (1–365) did at 0 mV in the absence of Ca^2+^ (Fig. 1o). However, the mutant CLCC1, including D25E, D181R, and D25E/D181R but not D152R/D153R and E175R/D176R, largely restored the currents in the presence of 10 mM Ca^2+^ at the *cis* side (Fig. 1n), suggesting that D25 and D181 are the key residues for the calcium inhibition on CLCC1 channel activity. Our Ca^2+^ dose response curve for the inhibition and the calculated IC_50_ (for WT, 1.09 ± 0.3 mM; D25E, 6.08 ± 3.2 mM; D181R, 8.51 ± 1.9 mM; D25E/D181R, 19.20 ± 7.4 mM) also supported the notion that D25 and D181 are the key residues (Fig. 1p). The IC_50_ for D152R/D153R (1.06 ± 0.4 mM) and E175R/D176R (2.83 ± 1.1 mM) suggested that these negatively charged residues are not involved in or less important than D25 and D181 for the inhibition.

To determine the Ca^2+^-binding affinity of the mutant CLCC1, we performed surface plasmon resonance (SPR)-based binding assay (Fig. 1q, r). Compared to the *K*_D_ value of the WT (1–365) (7.99 ± 0.69 mM), the *K*_D_ values of the mutant CLCC1, including D25E (10.47 ± 0.5 mM), E175R/D176R (11.29 ± 0.64 mM), D181R (12.14 ± 0.43 mM), and D25E/D181R (22.94 ± 5.1 mM), were significantly increased, suggesting that these mutations significantly impair Ca^2+^-binding affinity. Consistent with the strongest effect of the D25E/D181R mutant on relieving the Ca^2+^ inhibition, it also most significantly affects the Ca^2+^-binding affinity (Fig. 1s). Therefore, we conclude that D25/D181 are the key residues responsible for Ca^2+^-dependent inhibition of the channel open probability.

### CLCC1 maintains steady-state [Cl^–^]_ER_, [K^+^]_ER_, and ER morphology

**Fig. 2:**
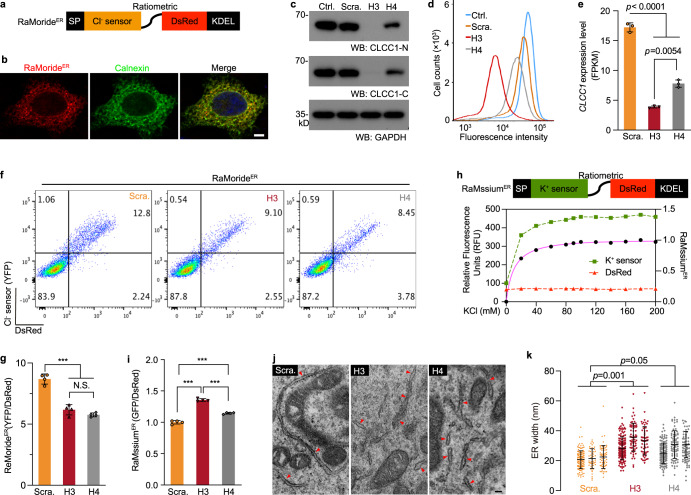
Loss of *CLCC1* increases steady-state [Cl^–^]_ER_ and [K^+^]_ER_ and leads to ER swelling. **a** Ratiometric Cl^–^ sensor (RaMoride^ER^) for ER [Cl^–^] ([Cl^–^]_ER_) measurement. An SP was tagged to the N-terminus of a previously reported Cl^–^ sensor,^27^ which was then fused to a monomeric DsRed as an internal control for the probe expression level and an ER retention signal (KDEL) at the C-terminal end, resulting in ratiometric Cl^–^ sensor (RaMoride^ER^) for [Cl^–^]_ER_ measurement. **b** ER localization of RaMoride^ER^. Immunofluorescence staining showing a prominent overlap of the DsRed fluorescence with Calnexin immunostaining signals. **c**–**e** Knockdown of *CLCC1* in 293FT cells infected with H3 and H4 shRNAs, as measured by western blot (**c**), FACS (**d**), and RNA-seq (**e**). Ctrl., MOCK control; Scra., scrambled shRNA; H3 and H4, shRNAs specific for *CLCC1*. **f**, **g** Steady-state [Cl^–^]_ER_ measured by RaMoride^ER^ in 293FT cells infected with the indicated shRNAs (**f**) and data summary (**g**). **h** Ratiometric K^+^ sensor (RaMssium^ER^) for ER [K^+^] ([K^+^]_ER_) measurement. An SP was tagged to the N-terminus of a recently reported K^+^ sensor,^28^ and fused to a monomeric DsRed and KDEL at the C-terminal end (upper). N-terminally His-tagged RaMssium^ER^ without SP was expressed in *E. coli* and the purified RaMssium^ER^ was diluted in isotonic solution with indicated [K^+^]. The ratiometric score of RaMssium^ER^ (purple) ranged from 0 to 1 (right *y* axis). **i** Steady-state [K^+^]_ER_ measured by RaMssium^ER^ in 293FT cells infected with the indicated shRNAs. **j** TEM images of 293FT cells infected with the indicated shRNAs. Ribosome-bound rough ER was marked by red arrows. **k** ER width was calculated from **j** and the data summary is shown. In **e**, **g**, **i** and **k**, values are presented as mean ± SD from at least three independent experiments or biological replicates; N.S., no significant difference, ***P < 0.001 by one-way ANOVA. Scale bars, 2 µm (**b**) and 50 nm (**j**).

To examine whether CLCC1 is involved in regulation of [Cl^–^]_ER_, we employed a previously optimized YFP Cl^–^ sensor that responds to Cl^–^ concentration change with super sensitivity and photostability.^27^ To create a ratiometric ER Cl^–^ sensor, we built a signal sequence, a DsRed internal control, and an ER retention motif into the Cl^–^ sensor, which we named RaMoride^ER^ (Fig. 2a). ER localization of RaMoride^ER^ was confirmed by its colocalization with ER-resident protein CALNEXIN (Fig. 2b). The ratio between YFP and DsRed signals responded correspondingly when extracellular [Cl^–^] ([Cl^–^]_Extra_) was switched from 140 mM to 100 mM or 0 mM (Supplementary information, Fig. S6a, b).

Consistent with the essential role of CLCC1 in vivo, we failed to generate a *CLCC1* knockout (KO) 293FT cell line by CRISPR/Cas9. Instead, we knocked down *CLCC1* with two individual shRNAs (H3 and H4) (Fig. 2c–e). Although the two shRNAs had different *CLCC1* knockdown efficiencies (for H3, 22.5% ± 1.04% of scrambled control; for H4, 45.25% ± 3.63% of scrambled control), both of them significantly increased steady-state [Cl^–^]_ER_ to a similar extent in comparison with scrambled shRNA control (Fig. 2f, g).

Given that the influx of K^+^ into ER lumen acts through a counter-ion mechanism during the internal Ca^2+^ release,^11^ we next measured steady-state [K^+^]_ER_ upon knockdown of *CLCC1*. To achieve this, we employed a newly reported K^+^ sensor^28^ and generated a ratiometric ER K^+^ sensor (RaMssium^ER^) (Fig. 2h), as we did for RaMoride^ER^. The RaMssium^ER^ responded well to a series of titrated K^+^ solutions in vitro (Fig. 2h) and a K^+^ ionophore, valinomycin (Supplementary information, Fig. S6c). With the RaMssium^ER^ probe, we detected significantly higher steady-state [K^+^]_ER_ in H3 and H4 groups compared to the scrambled control (Fig. 2i).

The concentration of electrically charged osmolytes, such as Cl^–^, inside a cell or intracellular membrane-bound organelle governs the volume of the compartment.^5,29^ Therefore, we asked whether the depletion of *CLCC1* changes ER volume. To this end, we collected 293FT cells carrying scrambled control or *CLCC1* shRNAs and applied transmission electron microscopy (TEM) (Fig. 2j, k). Enlarged and stubby ER morphology was documented in cells carrying the individual *CLCC1* shRNA. In contrast, ribosome-bound and tubule-like ER was shown in the scrambled control. To quantitatively reflect ER morphology, we measured ER widths in these three groups of cells. ER widths in the two individual *CLCC1* shRNA groups were significantly increased compared to the scrambled shRNA control. Therefore, we conclude that CLCC1 loss alters steady-state ER ion homeostasis and leads to enlarged ER.

### CLCC1 facilitates internal Ca^2+^ release

**Fig. 3:**
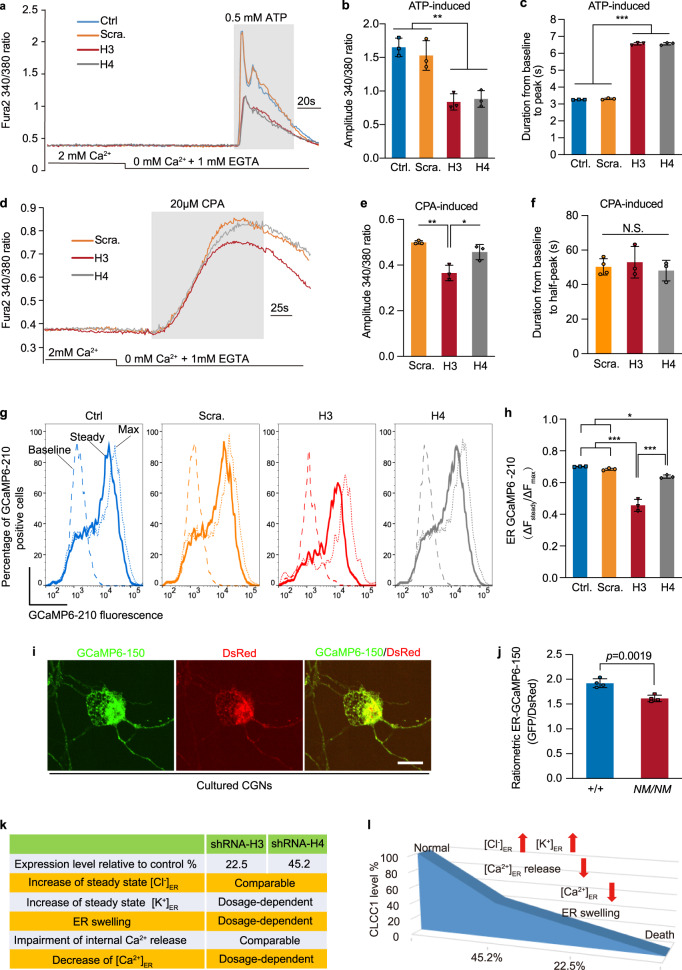
Depletion of CLCC1 impairs internal Ca^2+^ release and dosage-dependently reduces [Ca^2+^]_ER_. **a**–**c** 293FT cells infected with the indicated shRNAs were loaded with Fura-2 and stimulated with ATP in a calcium-free medium (gray rectangle). Representative Ca^2+^ release traces were averaged from at least 50 cells (**a**). The knockdown of *CLCC1* reduced the amplitude (**b**) but increased the time-to-peak (**c**) of ATP-induced Ca^2+^ release. **d**–**f** ER Ca^2+^ content was estimated by CPA-induced cytosolic Ca^2+^ rise in the calcium-free medium (gray rectangle) in 293FT cells infected with the indicated shRNAs. Shown are representative traces of CPA-induced calcium leak averaged from at least 50 cells (**d**) and data summary for the amplitude (**e**) and time-to-half peak (**f**) of CPA-induced cytosolic Ca^2+^ rise. **g**, **h** Steady-state [Ca^2+^]_ER_ in 293FT cells infected with the indicated shRNAs was measured by fluorescent signals of ER-GCaMP6-210, a previously reported low-affinity Ca^2+^ probe,^32^ by FACS. Baseline, 1 mM EGTA + 10 µM ionomycin; Steady, normal medium containing 2 mM Ca^2+^; Max, 10 mM Ca^2+^ + 10 µM ionomycin. The data summary (**h**) is from three independent experiments. ΔFsteady = (Fsteady – Fbaseline); ΔFmax = (Fmax – Fbaseline). Values are presented as mean ± SD. **i**, **j** Steady-state [Ca^2+^]_ER_ in the cultured CGNs infected with a ratiometric [Ca^2+^]_ER_ probe (ER-GCaMP6-150) was measured by fluorescent signal ratio of ER-GCaMP6-150 to DsRed (**i**). Data summary of [Ca^2+^]_ER_ between the indicated genotypes (**j**). **k**, **l** CLCC1 expression level and its relationships with ER ion homeostasis and morphology. In **b**, **c**, **e**, **f**, **h** and **j**, *n* > 150 cells pooled from three independent experiments. N.S., no significant difference, *P < 0.05, **P < 0.01, ***P < 0.001 by one-way ANOVA or *t*-test. In **i**, scale bar, 10 µm.

ER-localized ion channels have been proposed to control ER Ca^2+^ mobilization through a counter-ion mechanism.^13,14,16,17^ We then asked whether CLCC1, as an ER chloride channel, is involved in regulation of ER Ca^2+^ release. Knockdown of *CLCC1* by the two individual shRNAs markedly reduced internal Ca^2+^ release induced by ATP (Fig. 3a), which triggers ER Ca^2+^ release by generating IP3 that activates IP3Rs.^10^ Compared to mock control and scrambled shRNA, knockdown of *CLCC1* by the two individual shRNAs not only significantly reduced the amplitude, but also the rate (as reflected by the increase in time-to-peak), of ATP-induced Ca^2+^ release (Fig. 3b, c). Although the two shRNAs had different *CLCC1* knockdown efficiencies, they impaired the ATP-induced Ca^2+^ amplitude and rate to a similar extent.

Analysis of the Ca^2+^ release dynamics in individual cells revealed that *CLCC1* knockdown impaired ATP-induced Ca^2+^ oscillation (Supplementary information, Fig. S7a, b). Whereas less than 4.2% ± 1.0% of cells exhibited one ATP-induced Ca^2+^ spike in the scrambled shRNA group, the proportion was significantly more in the *CLCC1* knockdown groups (31.5% ± 4.0% for H3; 36.5% ± 8.5% for H4). However, the percentage of cells with Ca^2+^ spike number ≥ 3 was reduced in the *CLCC1* knockdown groups compared to that of scrambled control (Supplementary information, Fig. S7b). The impairment of ATP-induced Ca^2+^ release seems not to be caused by shRNA off-target effects, because the re-expression of full-length (WT) mCLCC1 restored the release damaged by H3 shRNA alone (Supplementary information, Fig. S8a, b). In contrast, the expression of mutant mCLCC1 lacking the ER lumen resident 2nd loop (Δ2nd loop) did not, suggesting that the 2nd loop of CLCC1 is crucial for its functions. Overexpression of full-length CLCC1 alone in the HEK293 cells has little effect on [Cl^–^]_ER_ and [K^+^]_ER_ but decreases the [Ca^2+^]_ER_ (Supplementary information, Fig. S9). Because the luminal side of CLCC1 has multiple Ca^2+^-binding sites, we speculate that overexpression of CLCC1 may bind to ER free Ca^2+^ close to ER membrane, resulting in the decreased [Ca^2+^]_ER_.

Next, we asked whether CLCC1 regulates internal Ca^2+^ release through RyRs, the predominant intracellular Ca^2+^ channels expressed in cardiomyocytes.^10^ To this end, we cultured cardiomyocytes from WT (+/+) and *NM2453* mutant (*NM*/*NM*) mice; the latter carries a hypomorphic mutant allele, an intracisternal A-particle retrotransposon (IAP) insertion into *Clcc1* that largely reduces CLCC1 protein expression^19^ (Supplementary information, Fig. S1a). The cultured cardiomyocytes were stimulated with caffeine, an agonist for RyR-mediated Ca^2+^ release. The RyR-mediated Ca^2+^ release was significantly reduced in *NM*/*NM* cardiomyocytes compared to WT (+/+) controls (Supplementary information, Fig. S7c), demonstrating that CLCC1 facilitates ER Ca^2+^ efflux through regulation of the release process per se rather than regulation of a particular type of Ca^2+^ channels.

### CLCC1 dosage is crucial for the maintenance of steady-state [Ca^2+^]_ER_

To examine whether the impaired Ca^2+^ release upon *CLCC1* knockdown results from a reduced ER Ca^2+^ load, we depleted the ER Ca^2+^ store with cyclopiazonic acid (CPA), an inhibitor of sarco/endoplasmic reticulum Ca^2+^-ATPase (SERCA).^30^ Knockdown of *CLCC1* by H3 but not H4 shRNA significantly reduced CPA-sensitive cytosolic Ca^2+^ rise (Fig. 3d–f), suggesting that depletion of ER Ca^2+^ content depends on CLCC1 dosage as H3 has higher knockdown efficiency than H4 shRNA (Fig. 2e).

Given that depletion of *CLCC1* increases ER volume (Fig. 2j, k), we next asked whether [Ca^2+^]_ER_ is also decreased. Because the [Ca^2+^]_ER_ ranges from 100 μM to 800 μM,^31^ we employed a previously reported low-affinity Ca^2+^ probe, ER-GCaMP6-210 ^32^, which correctly responded to CPA-induced internal Ca^2+^ depletion and follow-up ionomycin-mediated extracellular Ca^2+^ replenishment (Supplementary information, Fig. S10a). Compared to mock and scrambled shRNA controls, knockdown of *CLCC1* by both H3 and H4 shRNAs significantly decreased steady-state [Ca^2+^]_ER_ in cells expressing ER-GCaMP6-210 (Fig. 3g, h). The decrease caused by H3 shRNA was more severe than that by H4 shRNA, suggesting that depletion of *CLCC1* attenuates steady-state [Ca^2+^]_ER_ in a dosage-dependent manner.

To measure steady-state [Ca^2+^]_ER_ in the diseased neurons, we cultured cerebellar granule neurons (CGNs) from WT (+/+) and *NM*/*NM* mutant mice (Fig. 3i, j). We employed the low-affinity Ca^2+^ probe, ER-GCaMP6-150,^32^ and generated a ratiometric probe for [Ca^2+^]_ER_ measurement by fusing ER-GCaMP6-150 with DsRed, which responded well to CPA-induced internal Ca^2+^ depletion (Supplementary information, Fig. S10b). After infecting the cultured CGNs with the probe, we detected a significant decrease of [Ca^2+^]_ER_ in the mutant CGNs compared to the WT CGNs.

In summary (Fig. 3k, l), knockdown of *CLCC1* dosage-dependently increases steady-state [K^+^]_ER_ and ER volume and decreases steady-state [Ca^2+^]_ER_. In addition, the knockdown increases steady-state [Cl^–^]_ER_ and reduces internal Ca^2+^ release in a CLCC1 dosage-independent manner, suggesting that steady-state [Cl^–^]_ER_ and internal Ca^2+^ release are primarily affected by *CLCC1* knockdown.

### A conserved lysine (K298) is responsible for PIP2 facilitation of CLCC1 channel activity

**Fig. 4:**
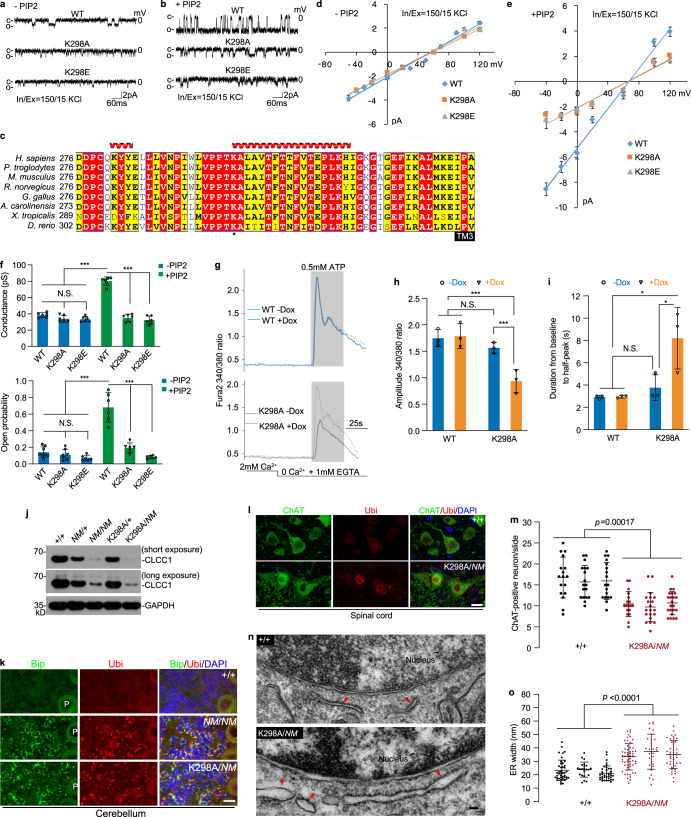
Mutation of K298, a PIP2-sensing residue for CLCC1 channel activity, impairs ER Ca^2+^ release and promotes ER swelling and neurodegeneration. **a**, **b** Single channel activities recorded after incorporating the purified WT, K298A, and K298E mutant mCLCC1 into the planar phospholipid bilayer. In **b**, the phospholipid bilayer contained 2% PIP2. **c** Alignment of sequences encompassing the 2nd loop of CLCC1 among different species. **d**, **e** *I-V* relationships in the absence (**d**) and presence (**e**) of PIP2 for WT mCLCC1 and its K298A and K298E mutants recorded from planar phospholipid bilayer in the asymmetric KCl solutions. **f** Data summary of slope conductance (upper) and channel open probability (*Po*) at 0 mV (lower) in the asymmetric KCl solutions. **g** An inducible lentiviral system was used to express WT mCLCC1 (WT) and its K298A mutant (K298A) in 293FT cells. ATP-induced Ca^2+^ release was measured in the calcium-free culture medium in Fura-2 loaded cells with (+Dox) or without (–Dox) induction. **h**, **i** Data summary of amplitude (**h**) and time-to-peak (**i**) of ATP-induced Ca^2+^ release under conditions shown in **g**. **j** Cerebellar expression of CLCC1 in the indicated genotypes. Short and long exposures were included for the same blot. GAPDH, loading control. **k** ER stress and misfolded protein accumulation documented by Bip and ubiquitin (Ubi) staining, respectively, in cerebella of *NM2453* homozygotes (*NM*/*NM*) and K298A and *NM2453* compound heterozygotes (K298A/*NM*). P, Purkinje cells. WT (+/+), negative control; *NM2453* homozygotes (*NM*/*NM*), positive control. **l**, **m** Ubiquitin-positive inclusions in ChAT-positive motor neurons in lumbar 4–5 spinal cords of K298A/*NM* mice. Representative images (**l**) and quantification of number of ChAT-positive motor neurons in the ventral horn (**m**) are shown. **n** TEM images of CGNs from WT (+/+) and K298A/*NM* mice. Red arrows indicate ribosome-bound rough ER. **o** Data summary of **n**. Mouse ages: 1.5 months (**j**, **k**); WT (+/ + ), 10 months, K298A/*NM*, 14 months (**l**, **m**); 3 months (**n**, **o**). Quantification: *n* = 4–20 (**d**–**f**); > 150 cells from three independent experiments (**h**, **i**); 14–18 sl**i**des (**m**) and > 25 granule cells (**o**) per mouse from three individual animals for each genotype. Scale bars, 20 µm (**k**), 10 µm (**l**), 50 nm (**n**). Values are presented as mean ± SD. N.S., no significant difference; *P < 0.05; ***P < 0.001, by *t*-test or one-way ANOVA.

As a necessary cofactor of many ion channels, PIP2, an acidic phospholipid of the cell membrane, has been implicated in regulating ion channel functions, including intercellular cation channels.^33–35^ To examine whether PIP2 affects CLCC1 channel activity, we included 2% PIP2 in the planar phospholipid bilayer. Interestingly, PIP2 significantly increased the single channel slope conductance (80.1 ± 2.5 pS) and the open probability (*P*_o_) of WT mCLCC1 (Fig. 4a, b). In addition, our macroscopic recordings also supported that PIP2 enhances the CLCC1 currents (Supplementary information, Fig. S11). Given that PIP2 regulates ion channels by binding to certain positively charged residues in the channel protein,^34,35^ we looked for positively charged residue(s) in CLCC1 and a positively charged lysine (K298) drew our attention (Fig. 4c). It lies in a consecutive row of six conserved residues — VPPTKA in the 2nd loop, which is required for CLCC1 facilitation of internal Ca^2+^ release (Supplementary information, Fig. S8). In addition, K298 is downstream of two proline residues, which usually present strong conformational rigidity, and lies at the beginning of a predicted α-helix.

We expressed and purified K298A mutant mCLCC1 and incorporated it into the lipid bilayer in the absence of PIP2, the mutant protein exhibited single channel activity with a slope conductance of 31.8 ± 3.7 pS, slightly lower than that of WT mCLCC1 (39.9 ± 2.3 pS) (Fig. 4a, d, and f). The *P*_o_ at 0 mV did not differ from that of WT mCLCC1 (Fig. 4a, f). Next, we mutated K298 to the negatively charged residue glutamate (K298E). Like K298A, K298E also had little effect on the channel activity in the absence of PIP2 (Fig. 4f). However, unlike WT mCLCC1 responsive to PIP2 (Fig. 4b, e), both K298A and K298E mutants abolished the responses, in terms of conductance and *P*_o_ (Fig. 4e, f). Therefore, we conclude that PIP2 facilitates CLCC1 channel activity and a conserved K298 in the 2nd loop is responsible for this facilitation.

### K298 is crucial for CLCC1 regulation of internal Ca^2+^ release

If K298 is functionally essential for CLCC1 channel activity, we wondered whether K298 is equally important for internal Ca^2+^ release. To examine this, we employed an inducible lentiviral system to stably express WT and K298A mutant mCLCC1 in 293FT cells in a controllable manner (Supplementary information, Fig. S12a). Expression of exogenous mCLCC1 proteins was induced after the application of doxycycline (Dox) (Supplementary information, Fig. S12b). Both the exogenous WT and K298A mutant mCLCC1 interacted with the endogenous hCLCC1 (Supplementary information, Fig. S12c), as shown by co-immunoprecipitation, supporting complex formation by exogenous mCLCC1 and endogenous hCLCC1 (Supplementary information, Fig. S2b). Induction of WT mCLCC1 did not alter the amplitude and rate of ATP-induced Ca^2+^ release (Fig. 4g–i). However, expression of K298A mutant mCLCC1 significantly suppressed such activities, as shown by the reduction in both the amplitude and rate when compared to un-induced (–Dox) cells or cells induced to express WT mCLCC1. In addition, induction of K298A mutant mCLCC1 expression, but not WT mCLCC1, decreased the ATP-induced Ca^2+^ oscillations (Supplementary information, Fig. S12d, e). These findings are all similar to those found in *CLCC1* knockdown cells (Fig. 3a–c; Supplementary information, Fig. S6a, b), suggesting a dominant-negative effect of the mutant protein in the CLCC1 channel function. Taken together, our findings reveal that a conserved K298 in the 2nd loop is functionally important for CLCC1 to regulate the internal Ca^2+^ release.

### K298A mutation promotes motor neuron loss and enlarges ER volume in vivo

To examine the in vivo effect of the conserved K298 residue, which is critical for PIP2 facilitation on CLCC1 channel activity and internal Ca^2+^ release, we generated a K298A knock-in mouse (Supplementary information, Fig. S13a, b). Although expression of K298A mutant mRNA and protein was confirmed by Sanger sequencing and mass spectrometry (Supplementary information, Fig. S13c–e), the expression level of K298A mutant protein was as low as that of the *NM2453* allele (Fig. 4j). Like *Clcc1* KO (Supplementary information, Fig. S14a, b), we failed to produce mouse homozygous for K298A (Supplementary information, Fig. S14c), indicating that K298 is a key residue for its essential function in vivo.

Compound heterozygotes with the *NM2453* and K298A mutations (*NM*/K298A) were viable but displayed severe body weight loss, hind leg weakness, trunk shaking, tail flagging, abnormal gaits, and ataxia phenotypes at as early as 3 months of age (Supplementary information, Video S1). The onset (~3 months of age) of these phenotypes is much earlier than that shown in the *NM*/*NM* mice (> 12 months of age),^19^ but slower than that of KO/*NM* mice (Supplementary information, Video S2), indicating that K298A is a partial loss-of-function allele. Like *NM*/*NM* mice, the *NM*/K298A compound heterozygotes displayed ER stress (Fig. 4k) and neuron degeneration in CGNs (Supplementary information, Fig. S13f). ER stress was also evident in hippocampal granule neurons in the compound heterozygous mice but not in *NM*/*NM* mice^19^ (Supplementary information, Fig. S13g). The severe motor impairment and hind leg muscle weakness prompted us to examine motor neuron pathologies in these compound heterozygous mice. Indeed, ubiquitin-positive inclusions in ChAT-positive motor neurons and their number loss, two key ALS pathologies, were evident in the mutant spinal cords (Fig. 4l, m).

As knockdown of *CLCC1* impairs ER ion homeostasis and leads to ER swelling (Fig. 2), we next asked whether dysfunction of CLCC1 impairs ER morphology in vivo. To this end, we examined the cerebella from WT and K298A/*NM* mice by TEM. Instead of ribosome-bound and tubule-like ER morphologies observed in WT CGNs, the mutant neurons harbored enlarged, stubby, and less ribosome-bound ER (Fig. 4n). Indeed, the ER width of mutant granule neurons was significantly increased compared to that of WT (Fig. 4o). Taken together, our findings demonstrate that disruption of channel function by the K298A promotes ER stress and motor neuron loss and enlarges ER volume in the diseased neuron in vivo.

### Rare genetic variants in *CLCC1* found in a Chinese ALS cohort

**Fig. 5:**
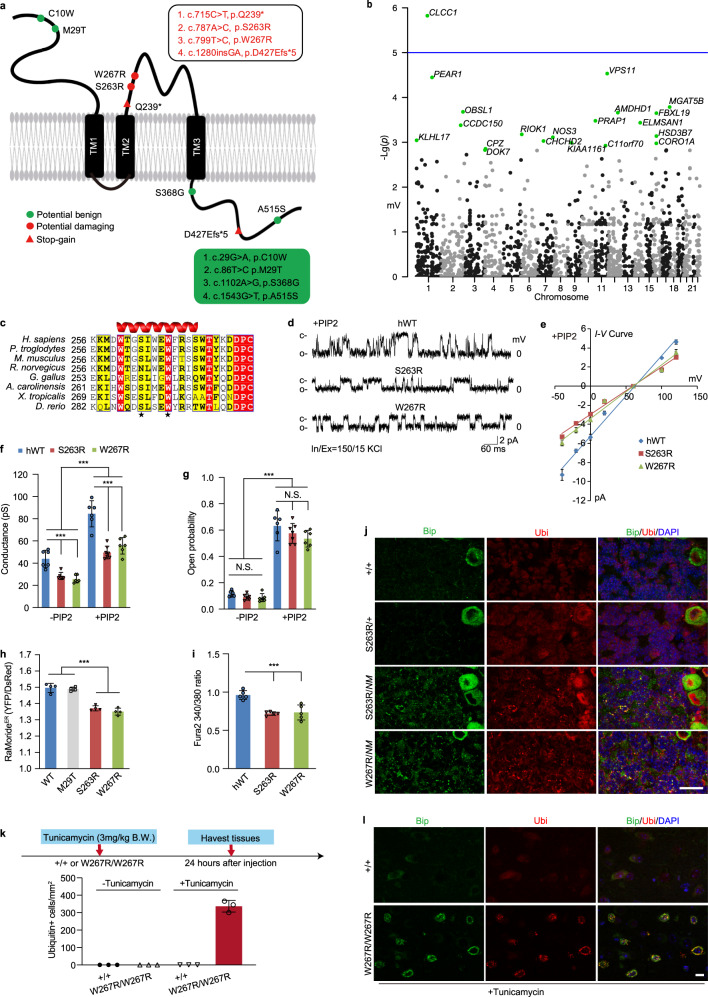
ALS-associated *CLCC1* mutations S263R and W267R impair CLCC1 channel functions and promote ER stress in vivo. **a** The nonsynonymous (colored circle) and stop-gain (red triangle) mutations of *CLCC1* were identified in a Chinese sporadic ALS cohort. The potential damaging mutations are labeled in red. **b** The Manhattan plot for an exome-wide rare variant burden analysis. The *P* value of *CLCC1* = 1.51 × 10^–6^, with OR = 5.72. **c** Protein sequence alignment of CLCC1 encompassing S263, W267, and neighboring residues. S263 and W267 are located in a predicted α-helix. **d**, **e** Single channel activities (**d**) and *I-V* relationships (**e**) recorded from planar phospholipid bilayers in the presence of PIP2 after the incorporation of purified hWT CLCC1 and its S263R, and W267R mutants, respectively. **f**, **g** Data summary of slope conductance (**f**) and channel open probability (*Po*) at 0 mV (**g**) in the absence and presence of PIP2. **h** Measurement of [Cl^–^]_ER_ in 293FT cells expressing WT or ALS-associated mutant CLCC1 by RaMoride^ER^. [Cl^–^]_ER_ was reflected by the ratio of YFP/DsRed fluorescent signals. Cells expressing WT or ALS-associated mutant CLCC1 were sorted by an engineered near-infrared fluorescent protein, miRFP670S,^70^ by FACS. **i** ATP-induced internal Ca^2+^ release was impaired by S263R and W267R mutants. Human WT (hWT) and S263 and W267 mutant CLCC1 were expressed in 293FT cells loaded with Fura-2. **j** ER stress and misfolded protein accumulation documented by Bip and ubiquitin (Ubi) staining in cerebella of compound heterozygous mice (S263R/*NM* and W267R/*NM*). S263R/+and WT (+/+) are negative for the phenotypes. **k**, **l** A diagram for tunicamycin challenge (upper, **k**). Animals with indicated genotypes were treated with one dose of tunicamycin (3 mg/kg body weight), an ER stress inducer. ER stress and accumulation of misfolded proteins were documented in W267R/W267R brains but not in that of WT (+/+). Data summary shown in (lower, **k**) and representative images shown in **l**. Values are presented as mean ± SD. N.S., no significant difference; ***P < 0.001, by one-way ANOVA. In **f** and **g**, *n* = 4–20; in **h**, *n* = 4 and in **i**, *n* = 5, > 50 cells per experiment; in **k**, 3 animals/genotypes, at least 4 slides/animal. In **j** and **l**, animal age, P30–P45; scale bar, 20 µm.

As lower motor neuron loss and its ubiquitin-positive inclusion are the key pathological features shown in ALS,^36^ we next asked whether the dysfunction of CLCC1 is relevant to motor neuron diseases. To this end, we performed whole-exome sequencing (WES) in a Chinese cohort (670 sporadic ALS patients and 1910 controls) and identified 8 rare variants in *CLCC1* in the patients, including 6 nonsynonymous and 2 stop-gain mutations (Fig. 5a; Supplementary information, Fig. S15 and Table S1). Among the mutations, the S263R and W267R mutations have not been found in the public databases nor in our controls (Supplementary information, Table S1). No mutations in known ALS-causing genes were detected in the patients carrying S263R or W267R mutations. Notably, two geographically and genetically unrelated patients with similar clinical phenotypes shared the same S263R mutation (Supplementary information, Tables S1, S2). Both mutations change Ser or Trp to Arg, suggesting that they perturb local steric hindrance and surface potential of the channel protein. A burden analysis^37^ was further carried out and revealed that *CLCC1* is associated with ALS (*P* = 1.51 × 10^–6^, with OR = 5.72), reaching suggestive significance (Fig. 5b).

### ALS-associated rare variants impair the channel activity and promote ER stress and protein misfolding in vivo

Evolutionarily, CLCC1 orthologues appear in vertebrates but not invertebrates and S263 and W267 are conserved across species (Fig. 5c). Furthermore, the 3-amino acid (aa) distance between S263 and W267 in a projected α-helix supports structural proximity and functional synergy in channel function and activity. To examine whether S263R and W267R affect CLCC1 channel activity, purified human wild-type (hWT), S263R, or W267R mutant CLCC1 proteins were incorporated into the lipid bilayer. In the absence and presence of PIP2, the slope conductance of both S263R and W267R was considerably lower than that of hWT (Fig. 5d–f). However, the *Po* of S263R and W267R was identical to that of hWT at 0 mV (Fig. 5g). S263R and W267R mutant CLCC1 also responded to PIP2, just like hWT, because PIP2 increased the slope conductance and *Po* of S263R and W267R mutant CLCC1 (Fig. 5f, g). To examine whether the ALS-associated rare variants affect [Cl^–^]_ER_, we expressed hWT, M29T, S263R, or W267R mutant CLCC1 in 293FT cells expressing RaMoride^ER^ (Fig. 5h). S263R or W267R mutant CLCC1, but not M29T mutant CLCC1, significantly decreased steady-state [Cl^–^]_ER_ compared to hWT, indicating that S263R and W267R are functionally detrimental alterations. Indeed, both S263R and W267R dramatically reduced ATP-induced internal Ca^2+^ release (Fig. 5i).

To examine the biological consequence of S263R and W267R in vivo, we generated S263R and W267R knock-in mouse lines (Supplementary information, Fig. S16a, b). Mice heterozygous for S263R and W267R were viable and fertile, and no obvious ER stress and protein misfolding were disclosed in S263R heterozygous mutant (S263R/+) cerebella (Fig. 5j) at ~1 month of age. However, Bip upregulation and ubiquitin-positive misfolded protein accumulation were documented in the cerebella of S263R/*NM* and W267R/*NM* mice, indicating that the ALS-associated rare variants promote ER stress and protein misfolding in vivo. We failed to harvest S263R/KO and W267R/KO pups, suggesting that the rare variants are functionally damaging in vivo, independent of the *NM2453* allele (Supplementary information, Fig. S16c, d).

Mice homozygous for S263R and W267R are viable and no obvious ER stress and protein misfolding was documented in the cerebellum, spinal cord, and thalamus. However, when we treated W267R/W267R mutant mice with a subdose of tunicamycin (3 mg/kg body weight),^38^ we observed Bip upregulation and misfolded protein accumulation in the thalamus and spinal cord of mutant mice but not in those of the WT mice (Fig. 5k, l; Supplementary information, Fig. S17), supporting the idea that the mutant neuron is more vulnerable to the ER stress challenge than the WT. A global proteasome-mediated degradation was evidenced by immune-reactive signals of Ub-K48, a major signal for target protein degradation by proteasome,^39^ in thalamus of the tunicamycin-treated animal (Supplementary information, Fig. S17b). Therefore, we concluded that two rare nonsynonymous mutations found in ALS, S263R and W267R, impair CLCC1 channel function and promote ER stress and protein misfolding in vivo.

### Dosage-dependent effect of CLCC1 in disease severity and cell-autonomous effect of CLCC1 in motor neuron loss and TDP-43 pathology

**Fig. 6:**
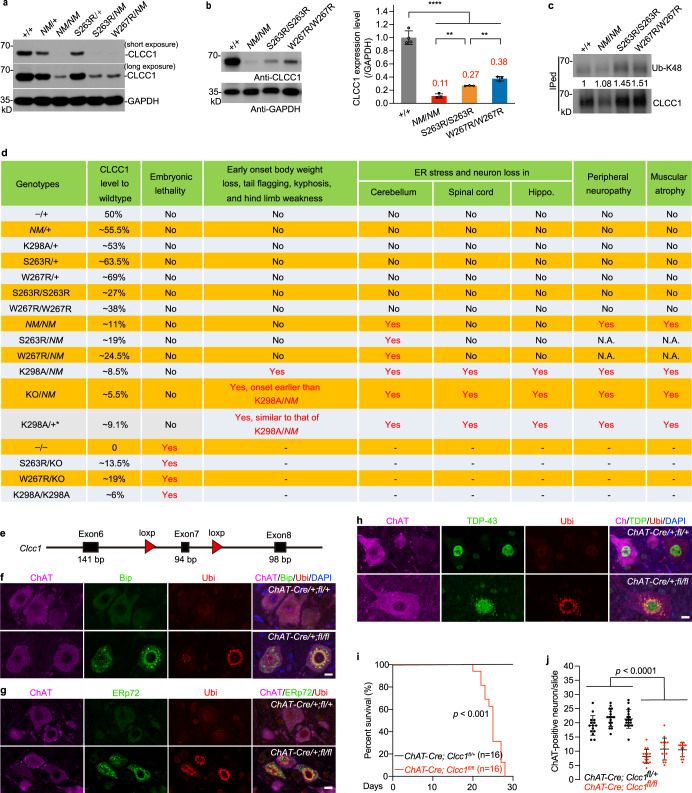
CLCC1 dosage dependence in severity of disease phenotypes in vivo and cell-autonomous effect of loss of CLCC1 function on motor neuron loss. **a**, **b** Cerebellar expression of CLCC1 in the indicated genotypes. Both S263R and W267R lowered the expression of CLCC1. Short and long exposures were included for the same blot (**a**). GAPDH, loading control. The data summary was shown in **b**. **c** K48-specific ubiquitination of S263R and W267R mutant CLCC1. **d** Phenotypic analysis for 5 available *Clcc1* alleles, including the previously reported *NM2453* (*NM*) and 4 new alleles reported in this study (KO, K298A, S263R, and W267R), which clearly demonstrate that the severity of mutant *Clcc1* phenotypes is CLCC1 dose-dependent and K298A, S263R, and W267R are loss-of-function alleles. **e** Construction of *Clcc1* conditional KO mouse. **f**–**j** KO of *Clcc1* in ChAT-positive motor neurons (*ChAT-Cre*;*Clcc1*^*fl*/*fl*^) leads to ER stress (**f**, **g**), TDP-43 pathology (**h**), early death (**i**), and motor neuron loss (**j**). *ChAT-Cre*;*Clcc1*^*fl*/+^ served as a negative control. In **b**, *n* = 4 for WT, *n* = 3 for others; in **j**, 14–18 slides per mouse from lumbar 4–5 spinal cords of three individual animals per genotype. Mouse ages in **f**, **g**, **h**, and **j**, P20–P25. Scale bars in **f**–**h**, 10 µm.

The ALS-associated rare variants decrease CLCC1 expression in the cerebellum (Fig. 6a, b), similar to K298A mutant CLCC1 protein, which is not as stable as WT CLCC1 (Fig. 4j; Supplementary information, Fig. S13d, e). Indeed, the S263R and W267R mutant CLCC1 undergo K48-specific ubiquitination (Fig. 6c), indicating proteasome-mediated degradation of mutant CLCC1 protein. However, the K298A, S263R, and W267R mutant CLCC1 are as stable as WT CLCC1 in a heterologous system, but the C-terminus is required for CLCC1 stability (Supplementary information, Figs. S12a–c, S18).

We then summarized CLCC1 expression levels and disease severity from the animals carrying one or two of the 5 *Clcc1* alleles, including the previously reported *NM2453* (*NM*) and 4 alleles generated in this study (KO, K298A, S263R, and W267R) (Fig. 6d). Because the *NM* allele is hypomorphic,^19^ it does produce WT CLCC1 protein but reduces the expression to ~5.5% of a WT allele (50%) (Fig. 6d). The KO/*NM* mice are viable and displayed similar phenotypes to K298A/*NM* mice, but the phenotype onset (1 month of age) is earlier than that (3 months of age) of K298A/*NM* mice, indicating that K298A is a partial loss-of-function allele (Fig. 6d; Supplementary information, Video S2). Although S263R and W267R impair CLCC1 expression to ~13.5% and ~19% of a WT allele, respectively, their expression levels are much higher than that (~3%) of K298A and that (~5.5%) of *NM* alleles (Fig. 6d).

In the K298A/+mice, we were surprised to find that ~10% (20/210, K298A/+*) animals appeared to exhibit severe phenotypes as early as 90.9 ± 24.5 postnatal days (Supplementary information, Fig. S19a, b and Video S3), reminiscent of the phenotypes shown in *NM*/K298A (Fig. 4; Supplementary information, Video S1). Curved spine (kyphosis) and muscular atrophy were also evident in K298A/+* but not in WT and K298A/+mice (Supplementary information, Fig. S19c, d). Because dosage of CLCC1 is critical for the mutant phenotypes, we examined CLCC1 expression in various tissues in these K298A/+* animals. As expected, CLCC1 expression level significantly decreased in these tissues compared to those of WT and K298A/+animals (Supplementary information, Fig. S20a, b). The decreased CLCC1 expression seems not to be explained by the decreased *Clcc1* mRNA level (Supplementary information, Fig. S20c).

Three major downstream pathways are involved in ER UPR, including PERK-eIF2α-ATF3, ATF6, and IRE1α-XBP1.^40^ For the PERK-eIF2α-ATF4 pathway, we detected upregulation of phospho-eIF2α and ATF4 in mutant cerebella, including the KO/*NM*, S263R/S263R, and W267R/W267R genotypes (Supplementary information, Fig. S21). For ATF6 and IRE1α-XBP1 pathways, we analyzed RNA-seq data from WT (+/+) and *NM*/*NM* cerebella. The known ATF6 downstream genes, including *Hsp90b1*, *Hspa5*, *Herpud1*, and *Ppp1r15a*, were all significantly upregulated in the *NM*/*NM* cerebella compared to that of WT (Supplementary information, Fig. S21a); however, there are no changes of the XBP1 downstream genes, including *Dnajb9*, *Prdm1*, *Syvn1*, and *Edem1*, between the two genotypes. These data suggest that dysfunction of CLCC1 leads to ER UPR mainly through PERK-eIF2α-ATF4 and ATF6 pathways. In addition, activation of proteasome-mediated protein degradation was evidenced by upregulation of genes involved in proteasome functions, global upregulation of ubiquitination, and K48-specific ubiquitination in the *NM*/*NM* and K298A/*NM* cerebella, respectively (Supplementary information, Fig. S22).

To gain insights into cell-autonomous or non-cell-autonomous effect of loss of CLCC1 function in motor neuron degeneration, we generated *Clcc1* floxed (fl) mouse (Fig. 6e) and crossed it with *ChAT-Cre* mouse,^41^ to knock out *Clcc1* in ChAT-positive motor neuron in spinal cord. ER stress was evidenced by upregulation of both Bip and ERp72 in ChAT-positive motor neurons in *ChAT-Cre*/+;*fl*/*fl* but not *ChAT-Cre*/+;*fl*/+ spinal cords (Fig. 6f, g). Misfolded protein accumulation was also evidenced by upregulation of ubiquitin in these *Clcc1* conditional KO neurons (Fig. 6f, g). Compared to nucleus-localized TDP-43 in *ChAT-Cre*/+;*fl*/+ motor neurons, cytoplasm-mislocalized and ubiquitin-positive TDP-43 (Fig. 6h), one of the pathological hallmarks of ALS,^42–44^ was documented in the conditional KO neurons. Indeed, all the *ChAT-Cre*/+;*fl*/*fl* animals died before P30 (Fig. 6i) with significant loss of motor neurons (Fig. 6j). Therefore, we conclude that the effect of loss of CLCC1 function in motor neuron loss is cell-autonomous.

## Discussion

Here, we characterized CLCC1 as a pore-forming component of an ER anion channel, the activity of which is inhibited by luminal Ca^2+^ but facilitated by PIP2. We link rare *CLCC1* mutations to ALS and demonstrate that the ALS-associated mutations impair CLCC1 channel activity, damage ER ion homeostasis, and promote ER stress in the brain, implying that disruption of ER ion homeostasis maintained by CLCC1 underlies the etiology of neurodegenerative disease.

During internal Ca^2+^ release, both Cl^–^ efflux through ER anion channel(s) and K^+^ influx through either TRIC family channels^11^ or RyRs/IP3Rs^8,15,45^ are indispensable for neutralizing membrane charge and balance of luminal osmolarity at the same time (Supplementary information, Fig. S23). Upon depletion/dysfunction of CLCC1, ER Ca^2+^ release was primarily impaired (Fig. 3), which we speculate was caused by reduced Cl^–^ through CLCC1, which no longer compensates for the membrane charge. Due to the complex nature of ATP-induced Ca^2+^ oscillation, involving IP3 metabolism, extracellular Ca^2+^ influx, and mitochondrial Ca^2+^ efflux,^46,47^ we do not know which cellular processes are majorly affected by the dysfunction/depletion of CLCC1. Therefore, doubling the amount of K^+^ influx through ER cation channels was needed to neutralize the membrane potential induced by Ca^2+^ release, which partially increases luminal osmolarity (Supplementary information, Fig. S23). As a consequence, increased steady-state [Cl^–^]_ER_ and [K^+^]_ER_ causes ER swelling, in turn enlarging ER volume and decreasing [Ca^2+^]_ER_, a high level of which is crucial for correct ER protein folding.^48^ Impairment of internal Ca^2+^ release seems to outperform the decrease of [Ca^2+^]_ER_/ER swelling (Fig. 3k, l), which explains the dosage-dependent effect of CLCC1 dysfunction/depletion on the severity of disease manifestations/ER stress in vivo (Fig. 6d). Given that CLCC1 is ubiquitously expressed,^19^ CLCC1 ER functions and their underlying mechanisms described here, including regulations on ER Ca^2+^ release, Ca^2+^ level, and morphology, could be applied to non-neuronal tissues and cell types.

CLCC1 shares little sequence similarity with any known ion channels, indicating that it belongs to a new channel family; therefore, we suggest renaming it ER anion channel 1 (ERAC1). Luminal Ca^2+^ inhibition on the channel activity prompts us to speculate a pre-inhibition mechanism in the resting state; when Ca^2+^ releases from ER, the local luminal [Ca^2+^] drops sharply, which in turn relieves the inhibition. CLCC1 channel activity is facilitated by PIP2 (Fig. 4), reminiscent of PIP2 positive regulation of TRIC channel activity.^34^ PIP2 does exist in ER/microsome which is almost 5% in ER and 20% in microsome fraction based on the different measurement methods, though its concentration is lower than that in plasma membrane (PM),^49–51^ and binds to TRICs, an ER counter-ion K^+^ channel.^34^ Amplification of both CLCC1 and TRIC channel conductance/*P*o by PIP2 may have biological relevance to large Ca^2+^ conductance of RyRs/IP3Rs during internal Ca^2+^ release.^8,45^ The above regulatory mechanisms suggest that the channel activity of CLCC1 is subject to multi-factor regulation, which is of great significance for an intracellular ion channel and deserves further study.

In this study, we observed that PIP2 increases CLCC1 conductance and channel *P*o (Fig. 4f); however, it is more common for PIP2 to change the *P*o of the given ion channels than conductance.^35,52–54^ Previously, PIP2 has been documented to dissociate from the regulatory module of TMEM16A resulting in ion-conducting pore collapse and subsequent channel desensitization.^55^ PIP2 also increased the single-channel conductance of TRPV1.^56^ To explain our results, we propose two possibilities. CLCC1 may have two open states, an intermediate open (subconductance) state and a fully open state, like TMEM16A.^55^ In the absence of PIP2, CLCC1 transits between the close state and intermediate open state. In the presence of PIP2, CLCC1 switches to a fully open state, which exhibits a higher *Po* and conductance. Alternatively, PIP2 may change the assembly property of pore-forming components and couple each unit’s opening, like TRIC channels.^34^ The previous study revealed the “chelating effect” of Ca^2+^ on PIP2^57^; therefore, it is plausible that Ca^2+^ “chelates” PIP2 in a steady state. During internal Ca^2+^ release, the local [Ca^2+^]_ER_ drops sharply, which relieves the Ca^2+^ “chelating effect” and results in PIP2-mediated facilitation on CLCC1.

CLCC1 channel activity is insensitive to voltage (Fig. 1d, e) but sensitive to DIDS (Supplementary information, Fig. S4), reminiscent of some early reported chloride currents recorded from SR/ER membrane preparation,^12,58–60^ but different from previously described CLCC1 currents.^20^ We cannot exclude the possibility that the previously reported currents recorded from microsome preparation^20,21^ are not CLCC1-mediated because many anion channel activities have been reported in the preparation.^12–17^ Alternatively, in this study, we interrogate the purified CLCC1 but not the overexpressed CLCC1 from microsome preparation.^20,21^ Many differences between the two settings, including protein modification, complex formation, the auxiliary protein component in the complex, and so on, may lead to the differences. Purified C350F mutant CLCC1 restored MTSET-mediated cytoplasmic side modulation on channel activity (Fig. 1h), suggesting that C350 is close to the CLCC1 anion permeation pathway.

In our ALS cohort, S263R was found in two unrelated patients, suggesting that *CLCC1* is an ALS risk gene. Physically, S263 and W267 are in close proximity (Fig. 5c), and functionally, both S263R and W267R lead to the biological consequences to a similar extent (Fig. 5d–i), suggesting they impair CLCC1 channel function probably through a similar mechanism. The rare variants are functionally damaging in vivo independent of the *NM* allele (Supplementary information, Figs. S17, S21b, c). However, we noticed that heterozygous or homozygous mice for ALS-associated S263R and W267R mutations did not display ALS-like phenotypes and pathologies (Fig. 6d) but decreased CLCC1 expression and activation of ER UPR (Fig. 6a, b; Supplementary information, Fig. S21), suggesting that a subthreshold ER stress occurs in these mice. We argue that environmental perturbation/stress may contribute to the disease development in these mutant animals. Indeed, W267R/W267R animals were sensitive to an ER stress challenge (Fig. 5), reminiscent of the neuron with ALS mutation in vivo more vulnerable to stress challenge than the WT.^61^

After summarizing the CLCC1 expression and disease phenotypes (Fig. 6d), we conclude that depletion of CLCC1 dosage-dependently increases the severity of disease manifestations in vivo (Fig. 6d). ALS-associated S263R and W267R mutant CLCC1 proteins undergo proteasome-mediated degradation in vivo (Fig. 6c). Although K298A was not found in our ALS cohort, ~10% K298A/+animals showed ALS-like phenotypes (Supplementary information, Fig. S19 and Video S3) and as little as ~9.1% (supposed to be ~53%) CLCC1 expression of WT (+/+) animals. Given that *Clcc1* mRNA levels between K298A/+* and K298A/+across various tissues are comparable (Supplementary information, Fig. S20c), we hypothesize that the mutant CLCC1 may dominant-negatively promote WT CLCC1 degradation by affecting homomultimer formation (Supplementary information, Figs. S2c, S18a). This observation also underscores a mechanism underlying channelopathy dominant-negatively caused by a loss-of-function mutation, which differs from haploinsufficiency. Phenotypically, K298A, S263R, and W267R are similar. All three mutations significantly impair CLCC1 channel activity (Figs. 4a–f, 5d–g) and reduce CLCC1 expression (Fig. 6d); three mutations promote the ER stress and neurodegeneration in vivo in the presence of *NM* allele (Fig. 6d); in the presence of *Clcc1* KO allele all three mutations lead to embryonic lethality (Fig. 6d). Given that S263R and W267R mutations appear dominant in our ALS cohort, heterozygosities for S263R and W267R may lead to ALS through a similar mechanism as evidenced in the K298A/+* mice. Therefore, further exploration of molecular pathways underlying mutant and WT CLCC1 protein degradation will provide important insights into the channelopathy mechanisms.

Purkinje cells express high levels of Calbindin-D28, which may confer strong calcium buffering capacity.^62^ This could explain the fact that Purkinje cells are not vulnerable to partial depletion of CLCC1, while the granule cells and motor neurons are.^19^ Another potential explanation for the different vulnerabilities of these neurons is the fact that the ER volume of Purkinje cells is larger than those of the granule neurons and motor neurons. A large ER volume is known to benefit ER ion handling.^16^ However, it is important to stress that the underlying mechanisms of neurodegeneration caused by CLCC1 dysfunction/depletion in cerebellar granule cells and motor neurons are similar. Indeed, cell-autonomous effect of loss of CLCC1 function on motor neuron loss and ubiquitin-positive and mislocalized TDP-43 (Fig. 6) links *CLCC1* dysfunction to common ALS pathologies and its underlying disease mechanisms.^42,63,64^ Dysfunction of RNA binding proteins (RBPs), including TDP-43, often leads to stress granule processing.^43,44^ It will be intriguing to further investigate the crosstalk between ER and membraneless organelles, like stress granule,^65^ and how dysfunctions in two cellular systems converge with the pathogenesis of ALS. It is also worthwhile to examine the crosstalk between ER and mitochondria and how mitochondria contribute to the underlying mechanisms of neurodegeneration caused by CLCC1 dysfunction because interaction between CLCC1 and a mitochondrial outer membrane microprotein was suggested to regulate UPR.^18^

## Materials and methods

### Protein expression and purification

The DNA fragments encoding mouse CLCC1-N (residues 12–200, NM_145543.2) and CLCC1-C (residues 355–539) were cloned into pET28A (Novagen) with an N-terminal 6× His tag or into pMAL-cRI with an N-terminal MBP (maltose binding protein, NEB) tag. The recombinant CLCC1 was expressed in BL21 derivative Rosetta (DE3) at 37 °C overnight. After ultrasonic cell disruption, the recombinant proteins in the soluble fractions were purified by Ni-NTA resin (Qiagen) or amylose resin (NEB) and dialyzed overnight in 10 mM PBS solution. For the insect expression system, the full-length mouse and human *CLCC1* (WT, C350F, K298A, and K298E, S263R and W267R) were cloned into pFastbac-1 (Invitrogen) with a C-terminal His_10_ tag. The bacmids were extracted from DH10 Bac bacteria and transfected into Sf9 insect cells, which were grown in SFX-Insect cell culture medium (GE Healthcare) at 26 °C to generate and amplify baculovirus (Bac-to-Bac system, Invitrogen). About 200 mL of High Five insect cells (1 × 10^6^ cells/mL SIM HF culture medium, Sino Biological Inc.) were infected by 4 mL baculovirus to express the recombinant proteins. The infected High Five cells were harvested 48 h after infection and homogenized in the TBS lysis buffer (50 mM Tris-HCl, pH 7.4, 150 mM NaCl, 1% *n*-Dodecyl-β-D-Maltopyranoside (DDM, Inalco), and protease inhibitor cocktail, including 2 μg/mL pepstatin A, 4 μg/mL aprotinin, 10 mg/mL 4-(2-Aminoethyl) benzenesulfonyl fluoride hydrochloride, 4 μg/mL bestatin, 4 μg/mL E-64, 4 µg/mL leupeptin, and 1 mM phenylmethane sulphonylfluoride) on ice for 30 strokes with Dounce homogenizer, and then rotated for additional 30 min. The cell debris was removed by centrifugation at 30,000× *g* for 1 h. The supernatant was harvested carefully, followed by addition of 10 mM imidazole, and incubated with Ni-NTA resin (Qiagen). The resin was washed with TBS buffer containing 0.05% DDM and 100 mM imidazole. The proteins were eluted from beads with TBS buffer containing 0.05% DDM and 300 mM imidazole. The resulting proteins were treated with 2 mM DTT and incubated on ice for 30 min. The final concentrated proteins were further purified by size-exclusion chromatography (Superose 6 Increase, GE Healthcare) in the TBS buffer containing 0.025% DDM and 2 mM DTT. Standard molecular weight markers shown in the user manual (GE Healthcare) were used to estimate the size of protein complex. The peak fractions were collected, frozen in liquid nitrogen, and stored at –80 °C for electrophysiology studies.

The DNA fragments encoding WT human CLCC1-N (residues 1–365) or with mutations, including D25E, D152R/D153R, D175R/E176R, D181R, and D25E/D181R, were cloned into pET28A (Novagen) with a 6× His tag. The recombinant CLCC1 was expressed in BL21 derivative Rosetta (DE3) at 37 °C. The cells were harvested 16 h after IPTG (1 M) induction and the protein purification procedure was similar to that of mCLCC1 described above.

### Planar bilayer lipid membrane recording

Lipid bilayers formed across an aperture 0.2 mm in diameter in a delrin cup, with a mixture of phosphatidylcholine (PC), phosphatidylserine (PS) (Avanti Polar Lipids) and phosphoethanolamine (PE) (Lipoid) in a weight ratio of 1:2:2. The lipids were dissolved in *n*-decane (Sigma) at a concentration of 50 mg lipid/mL *n*-decane. All solutions were buffered by 10 mM HEPES (pH 7.4). The lipid bilayer separated the *cis* (In) solution from the *trans* (Ex) solution (1.0 mL each) and the purified WT CLCC1 and its mutant variants were added to the *cis* side of a lipid bilayer membrane. The purified proteins were added at *cis* side and the membrane potential represents the voltage potential at *trans* side. The single channel currents were recorded by adding 3.5 μL of 1.8 mg/mL protein to the *cis* side in asymmetric KCl solution (In/Ex, 150/15 mM) at indicated voltages. The square step-like multiple-channel currents were recorded by adding 5 μL of 1.8 mg/mL protein to the *cis* side in asymmetric KCl solution (In/Ex, 150/15 mM) at 0 mV. The macroscopic currents were recorded by adding 35.0 μL of 1.8 mg/mL protein. The membrane potentials were held at +60 mV and then stepped to a prepulse from –40 mV to +100 mV with 20 mV increments for 3 s to elicit currents. The channel currents were recorded in a voltage-clamp mode using a Warner BC-535 bilayer clamp amplifier (Warner Instruments) filtered at 1 kHz, 25 °C. The currents were digitized using pCLAMP 10.4 software (Molecular Devices). The single-channel conductance was determined by fitting to Gaussian functions. Opening times < 0.5–1.0 ms were ignored. The theoretical equilibrium potential was calculated using the Nernst equation. The open probability *P*_o_ = t/T, where t is the total time that the channel is observed in the open state and T is the total recording time. The ion selectivity was calculated using the Goldman-Hodgkin-Katz flux equation:

$$E_{rev} = - \frac{{RT}}{{zF}}\ln \frac{{P_A\left[ {{{{{{{\mathrm{A}}}}}}}} \right]_o}}{{P_B\left[ {{{{{{{\mathrm{B}}}}}}}} \right]_i}}$$

To examine the inhibitory effects of MTSET and DIDS, a certain amount of stocks of the two drugs were added into the either *cis* or *tran*s chamber using a pipette.

### SPR assay

Biacore T200 instruments (Cytiva) were used to detect the binding affinity of Ca^2+^ to CLCC1 protein with or without mutations via SPR. Briefly, the protein was immobilized on the surface of the CM5 chip by using an amine-coupling approach at a flow rate of 10 μL/min in 10 mM acetate buffer (pH 4.0). The sensor surface was activated with a 7 min injection of the mixture of 50 mM N-hydroxysuccinimide (NHS) and 200 mM 1-ethyl-3-(3-dimethylamino propyl) carbodiimide (EDC). Then 60 μg/mL of protein was injected to reach the target level of 16,000 RU and the surface was blocked with 1 M ethanolamine (pH 8.5). A series of concentrations of Ca^2+^ (typically 9.76 μM, 19.53 μM, 39.06 μM, 78.13 μM, 156.25 μM, 0.31 mM, 0.63 mM, 1.25 mM, 2.50 mM, 5.00 mM, 10.0 mM, 20.0 mM) were injected into the flow system and analyzed for 90 s, and the dissociation was 90 s. All binding analyses were performed in 20 mM Tris buffer (pH 7.5) at 25 °C. Before analysis, double reference subtractions were made to eliminate bulk refractive index changes, injection noise, and data drift. The binding affinity was determined by fitting to a Langmuir 1:1 binding model within the Biacore Evaluation software (Cytiva).

### The generation and purification of CLCC1 polyclonal antibodies

To generate CLCC1 polyclonal antibodies, the purified mCLCC1-N (residues 12–200) and mCLCC1-C (residues 355–539) tagged with MBP were used to immunize the rabbits (SPF Japanese white rabbit). The subcutaneous inoculation was given once two weeks at least 3 times (0.1 mg antigen in complete/incomplete Freund’s Adjuvant/rabbit, Sigma). The rabbit anti-serum was collected and purified by NHS-activated Sefinose beads conjugated by His-tagged mCLCC1-N or mCLCC1-C. The resulting antigen–antibody complexes were washed with PBS containing 0.15% Triton X-100 to reduce non-specific binding. The polyclonal antibodies with high affinity were eluted from the Sefinose beads with 50 mM glycine (pH 2.5), and neutralized to pH 7.4 immediately with Tris-HCl buffer.

### Microsome isolation, rough ER isolation, and protease digestion

The microsome isolation was performed as previously described with slight modifications.^24^ The brains and the livers of WT mice (0.5 mg tissue per preparation) were disrupted using a Dounce homogenizer for 30 strokes in a working buffer (225 mM mannitol, 75 mM sucrose, and 30 mM Tris-HCl, pH 7.4) on ice. The nuclei and unbroken cells were removed by centrifugation at 1000× *g* for 10 min. The supernatants containing the PM and the ER fractions were harvested by further centrifugation at 10,000× *g* for 10 min. The final pellet was collected at 25,000× *g* for 30 min and resuspended in the working buffer. Protease digestion assay was performed as previously reported with some modifications.^66^ In brief, the isolated microsome vesicles were incubated at 25 °C for 30 min with trypsin (Sigma). The digestion was performed in the absence or presence of 0.1% (v/v) Triton X-100 and stopped by adding anti-trypsin inhibitor for 10 min on ice. To isolate the rough ER, the supernatants containing the PM and the ER fractions were harvested by further centrifugation at 10,000× *g* for 10 min. CaCl_2_ solution was added to the microsome dropwise with constant stirring to the final concentration (7 mM) and continued stirring for 15 min. Then, the microsome was centrifuged at 8000× *g* for 10 min to collect the pellet, which mainly contains rough ER fraction. All centrifugation steps were executed at 4 °C.

### Chemical crosslinking experiments

Protein crosslinking experiments were performed according to the user instruction (Thermo Fisher Scientific). Briefly, for in vitro crosslinking, the purified N- and C-CLCC1 were incubated with DSS (Thermo Fisher Scientific) for 30 min at 25 °C, followed by adding quenching buffer (1 M Tris-HCl, pH 8.0). We set the DSS concentration gradients ranging from 0 mM to 1 mM. For in vivo crosslinking, 293FT cells were harvested and washed with PBS twice. The resulting cells were incubated with different concentrations of DSS at room temperature, and then treated with the quenching buffer.

### ER [Cl^–^], [K^+^], and [Ca^2+^] measurement

For ER steady-state [Cl^–^] measurement, we modified a previously reported Cl^–^ probe,^27^ by adding an SP and an ER retention signal KDEL and fusing it with a monomeric DsRed. The resulting ratiometric ER Cl^–^ probe was named RaMoride^ER^. Naïve 293FT or *CLCC1* knockdown cells were transfected with RaMoride^ER^ and then washed with HBSS buffer without calcium and magnesium. To validate RaMoride^ER^, we suspended the cells with buffer containing 0.6 mM MgSO_4_, 38 mM NaCl, and 100 mM KCl (20 mM HEPES, pH 7.4), or corresponding extracellular [Cl^–^] ([Cl^–^]_Extra_) by replacement of Cl^–^ by gluconate. To estimate ER steady-state [Cl^–^], the cells were suspended in HBSS buffer containing 2 mM Ca^2+^ and 140 mM Cl^–^.

To characterize the [K^+^] sensor in vitro, the K^+^ binding protein (mNG-Ec-Kbp-E12A), also named KRaION2 by a previous report,^28^ was fused with monomeric DsRed and subcloned into pBAD vector with an N-terminal His tag. The expression was induced by the application of 0.06% arabinose overnight. The purified ratiometric K^+^ sensor was used to measure the binding ability to K^+^ by Varioskan Flash (Thermo) in a solution containing 25 mM Tris-HCl (pH 7.0), 75 mM NaCl, and sodium gluconate, the concentration of which was in accordance with that of KCl we added to keep the osmolarity unchanged. To validate the ratiometric potassium sensor in 293FT cells, an N-terminal SP and a C-terminal KDEL were added and the resulting probe was named RaMssium^ER^. The cells expressing RaMssium^ER^ were suspended and the fluorescence was measured by FACS in a PBS solution with valinomycin (Cayman, 10 mM), a highly selective potassium ionophore.

For ER steady-state [Ca^2+^] measurement, we transfected naïve 293FT or *CLCC1* knockdown cells with a previously reported ER-targeted low-affinity calcium probe.^32^ The transfected cells were washed with HBSS buffer without calcium and magnesium and then suspended with the following buffers separately: the HBSS solution containing 10 µM ionomycin (Beyotime) and 1 mM EGTA for baseline (Fbaseline); the HBSS solution with 2 mM CaCl_2_ for steady-state (Fsteady); the HBSS solution with 10 mM CaCl_2_ and 10 µM ionomycin for saturating the probe (Fmax). The relative ER steady-state [Ca^2+^] was estimated by ΔFsteady (Fsteady – Fbaseline) divided by ΔFmax (Fmax – Fbaseline). The fluorescent signals from individual cells were collected by LSRFortessa flow cytometer (BD Biosciences). For Cl^–^ sensor, K^+^ sensor, and ER-GCaMP6-210, we employed the FITC channel (488 nm); for DsRed, we employed PE channel (561 nm); for miRFP670S, we employed APC channel (647 nm). Data were analyzed by FlowJo X. The cells were treated with 7-AAD (BioLegend) or DAPI (Beyotime) to exclude the dead cells.

For a ratiometric ER Ca^2+^ sensor, we employed a previously reported ER-GCaMP6-150 and adopted a similar strategy for the generation of RaMssium^ER^. The ER-GCaMP6-150 and DsRed were subcloned into pLJM-EGFP (Addgene plasmid # 19319) for lentivirus packaging. To validate the ratiometric ER Ca^2+^ sensor, we transiently expressed it in 293FT cells treated with CPA and then sorted the cells for measurement by FACS. The cultured neurons were transduced with the lentiviral probe and the fluorescence was measured by confocal imaging.

### Primary neuron culture

The glass-bottom culture dish was pretreated with poly-D-lysine (Thermo) overnight. The cerebellum of mice at P5 was dissociated and digested by 0.25% trypsin for 12 min and 30 μg/mL DNAse I (Sigma) for additional 3 min at 37 °C. Dulbecco’s modified Eagle’s medium (DMEM) containing 10% fetal bovine serum (FBS) was added to stop the digestion and then the undigested tissue was removed using a 70 μm cell strainer (BD). The neurons were resuspended in DMEM medium containing 10% FBS and seeded into the culture dish for 2 h. After that, the medium was removed and Neurobasal-A medium containing B-27 supplement and 125 mM KCl was added. One day later, the lentivirus was added to the medium. We performed confocal imaging two days after the infection (Zeiss LSM780). The endothelial and glial cells were distinguished from neurons by their shapes.

### TEM

Mice at P30 were perfused by 0.1 M phosphate buffer (PB, pH 7.4) at room temperature, and then fixed by fixation solution (FS, 4% paraformaldehyde (PFA) (W/V) in PB) and by 2.5% glutaraldehyde in FS at 4 °C overnight. Similar regions of the cerebellum were cut into 200 μm for embedding, which is performed at the Center for Biomedical Analysis of Tsinghua University. The images were captured by Tecnai Spirit electron microscopy.

### Lentivirus-medaited shRNA knockdown and inducible expression system

Lentiviruses were produced by co-transfecting 293FT cells with transfer constructs, pMD2.G and psPAX2, by linear PEI (MW 25,000, Polysciences). The medium containing lentivirus without debris was concentrated by centrifugation at 20,000 rpm for 2 h and resuspended in PBS. For the generation of the stable cell line carrying human *CLCC1* shRNA (MissionRNAi, Sigma), the 293FT cells were selected with 1 µg/mL puromycin. For the construction of the inducible expression system, we modified the pCW-cas9 (pCW-Cas9, Addgene #50661), in which the *Cas9* was replaced by our target genes. After 48-h drug resistance selection, the cells were maintained in a medium containing appropriate antibiotics and used within one week. The knockdown efficiency and inducible expression of proteins of interest were examined by western blot. For exogenous WT and mutant CLCC1 expression induction, 1 μg/mL Dox (Sigma) was applied in the culture medium.

### Cell culture and calcium imaging

293FT and HeLa cells were maintained in DMEM supplemented with 10% heat-inactivated FBS and 1% penicillin/streptomycin (GE Healthcare). The primary cardiomyocyte culture was performed as previously reported.^67^ Briefly, the hearts from P2 neonatal mice were dissected and minced in the Ca^2+^- and Mg^2+^-free PBS supplemented with 20 mM BDM (Sigma). The chopped tissues were digested in PBS containing 0.125% (w/v) trypsin at 4 °C for 2 h followed by the digestion of 0.5% collagenase I (Sigma) at 37 °C for 30 min. After the digestion, the cardiomyocytes were seeded on gelatin (Sigma)-coated coverslips and in DMEM/F12 medium containing 10% FBS. After 48 h, the cardiomyocytes showed spontaneous beating, which were used in calcium imaging experiments. For calcium imaging, the 293FT cells or cardiomyocytes seeded on the coverslips were loaded with the ratiometric Ca^2+^ indicator (Fura-2 AM, Thermo Fisher Scientific) in Krebs-Ringer-HEPES (KRH) buffer (25 mM HEPES, pH 7.4, 125 mM NaCl, 6 mM glucose, 5 mM KCl, 1.2 mM MgCl_2_) supplemented with detergent Pluronic F-127 (Thermo Fisher Scientific). After 30-min loading at room temperature in dark, the coverslip was washed twice with KRH buffer and then subjected to calcium imaging in a perfusion chamber on an inverted Nikon TiE microscope with 20× Fluar objective. The Metafluor Program software (Molecular Devices) was used to monitor and calculate the real time changes of calcium concentration in the cytoplasm.

### Western blot, immunoprecipitation, and immunostaining

For western blot and immunoprecipitation, the cultured cells or tissues were lysed in the TBS lysis buffer (50 mM Tris-HCl, pH 7.4, 150 mM NaCl, 1% DDM, and protease inhibitor cocktail). After incubation for 20 min on ice, the cell debris was removed by centrifugation at 13,000× *g* for 5 min. For western blot, the supernatant was boiled with 2× SDS loading buffer and the proteins were separated on SDS-PAGE gel and transferred to PVDF membrane (GE Healthcare) using standard protocol. The blot was incubated with the primary antibody overnight at 4 °C, and then HRP-conjugated secondary antibody at room temperature for 60 min. For immunoprecipitation assay, the Dynabeads (Invitrogen) were used to capture the tagged target proteins. The beads were washed with the TBS lysis buffer, pre-incubated with the primary antibody at room temperature for 20 min, and then incubated with the supernatant of the cell lysate at 4 °C for at least 3 h or overnight. The beads were washed five times with washing buffer (50 mM Tris-HCl, pH 7.4, 150 mM NaCl, 0.025% DDM, and protease inhibitor cocktail). The immunoprecipitated proteins were eluted by 2× SDS loading buffer at 95 °C for 5 min. For cultured cell immunostaining, the cultured HeLa cells were fixed with 4% PFA (W/V) and permeabilized by 0.3% Triton X-100 in PBS for 10 min. The fixed cells were blocked with blocking buffer (PBS with 3% BSA), stained with primary antibody overnight at 4 °C, and then incubated with secondary antibody for 1 h at room temperature. For tissue immunostaining, the PFA-fixed paraffin-embedded sections were deparaffinized with standard protocol as described previously.^19^ For antigen retrieval, the section was boiled in the sodium citrate buffer (10 mM sodium citrate, pH 6.0) and cooled to room temperature. After antigen retrieval, the sections were blocked with the blocking buffer and stained with the primary and secondary antibodies. For antibodies, the following primary antibodies were used, including anti-Calnexin (1:5000, WB, Proteintech), anti-RCAS1 (1:1000, WB, Cell Signaling Technology), anti-ATP5A1 (1:5000, WB, Proteintech), anti-Lamin B1 (1:10,000, WB, Proteintech), anti-FLAG (1:5000, clone 3B9 mouse, WB, Abmart), anti-Myc (1:5000, clone 19C2 mouse, WB, Abmart), anti-tubulin (1:10,000, clone B-5-1-2 mouse, WB, Sigma), anti-calmodulin binding protein (1:2000, rabbit, WB, Millipore), anti-Bip (1:300, rabbit, IF, Abcam), anti-ubiquitin (1:200, P4D1 mouse, IF, Cell Signaling Technology), anti-ubiquitin-K48 (1:200, IF, 1:1000, WB, Abcam), anti-ATF4 (1:1000, WB, Abcam), anti-eIF2α and phosphorylated eIF2α (1:1000, WB, Cell Signaling Technology), anti-TDP-43 (1:200, IF, Proteintech), anti-ChAT (1:200, IF, Millipore), anti-ERp72 (1:200, IF, Enzo), anti-NeuN (1:200, IF, Millipore), anti-His (1:1000, rabbit, WB, Cell Signaling Technology), and anti-GAPDH (1:5000, 14C10 rabbit, WB, Cell Signaling Technology). For secondary antibodies, we used Alexa-conjugated secondary (488, 555, 647) antibodies (Life Technologies; Molecular Probes) at 1:500 and HRP-linked secondary antibodies (GE Healthcare) at 1:5000. For quantification of relative CLCC1 expression level, protein samples from the same brain regions were loaded on, run in one gel, and transferred to one PVDF membrane. After being probed by CLCC1 and GAPDH antibodies, the chemiluminescence signals of the bands were captured on an X-ray film (Kodak) or by Tanon 5200 Multi system. The integrated density of the GAPDH band was divided by that of CLCC1 from the same lane and then these ratios were normalized to that of WT mice. To ensure the reliable comparison, the pictures with any over-exposed band could not be used for quantification. The digital file was analyzed by ImageJ.

### Generation of the knock-in and *Clcc1* floxed mouse and genotyping

For the generation of the knock-in mouse line, we synthesized the DNA oligo which carried the target mutations. The gRNAs (ttggttggttccaccaacaaAGG for K298A, tggattggactggaagtctcTGG for S263R, and ttggcatgggtcatccttatAGG for W267R, PAM sites capitalized) were generated by in vitro transcription (Invitrogen). The donor DNA oligo, gRNA, and *Cas9* mRNA were injected into C57BL/6J embryos. The injected embryos were transferred into the oviduct ampulla of the pseudo-pregnant ICR (JAX, Stock# 009122) female recipients. The right genotype offsprings were backcrossed with C57BL/6J for at least three generations to establish the line. For genotyping the K298A knock-in mouse, the gDNA PCR (forward primer: ggcacagtcaaaaccaaactgatcttg and reverse primer: gagcctaaaaccaaagaccagagc) products were digested with *Msp*A1l (NEB). Primers for the S263R knock-in mice are ggatttgcgttcccagctcggtt (forward) and tccgtcccttttaactttgaggcag (reverse), and for the W267R knock-in mice are gtgggcacagtcaaaaccaaactga (forward) and gagcctaaaaccaaagaccagagca (reverse). The gDNA PCR products were confirmed by Sanger sequencing. The animal facility at Tsinghua University has been fully accredited by the Association for the Assessment and Accreditation of Laboratory Animal Care International since 2014. All animal protocols were approved by the Institutional Animal Care and Use Committee at Tsinghua university based on Guide for the Care and Use of Laboratory Animals (8th Edition, NHR). *Clcc1* floxed mice were generated by Cyagen (China). Two loxP sites were inserted into the introns 6 and 7 by CRISPR/Cas9, respectively. The founders were backcrossed with C57BL/6J mice for at least three generations to reduce the off-target effect.

### Tunicamycin treatment

Tunicamycin was diluted in 150 mM glucose at 0.3 μg/μL from the 10 mg/mL stock solution. As previously reported, the mice at P30 received one dose of administration subcutaneously at 3 μg/g body weight.^38^ All the mice were sacrificed 24 h later after injection, and the brain and spinal cord were dissected and fixed in 4% PFA/PBS solution.

### Molecular biology

The following sequences of CLCC1 homologues from different vertebrate species were obtained from the NCBI GenBank: *Homo Sapiens* (NM_001048210.2), *Mus musculus* (NM_145543.2), *Pan troglodytes* (XM_009426847.2), *Rattus norvegicus* (NM_133414.1), *Gallus gallus* (XM_422186.5), *Anolis carolinensis* (XM_003223596.3), *Xenopus tropicalis* (XM_002932173.4), *Danio rerio* (XM_002667211.5). The alignment result was done by using the Clustal W program and reported from http://espript.ibcp.fr/ESPript/cgi-bin/ESPript.cgi.^68^

### Mass spectrometry

Brain lysates of K298A/*NM* mice were applied for immunoprecipitation with CLCC1-C antibody. Gel bands between 55 kD and 100 kD from the immunoprecipitation were excised for in-gel digestion, and the WT and K298A CLCC1 small peptides were identified by mass spectrometry (MS) as previously described.^69^ Briefly, proteins were disulfide reduced with 25 mM dithiothreitol (DTT) and alkylated with 55 mM iodoacetamide. In-gel digestion was performed using sequencing grade-modified pepsin in 1% fomic acid at 4 °C for 30 min. The peptides were extracted twice with 1% trifluoroacetic acid in 50% acetonitrile aqueous solution for 30 min. For LC-MS/MS analysis, peptides were separated by Thermo-Dionex Ultimate 3000 HPLC system. The analytical column was a homemade fused silica capillary column (75 μm ID, 150 mm length; Upchurch, Oak Harbor, WA, USA) packed with C-18 resin (300 A, 5 μm; Varian, Lexington, MA, USA). Mobile phase A consisted of 0.1% formic acid, and mobile phase B consisted of 100% acetonitrile and 0.1% formic acid. MS/MS spectra from each LC-MS/MS run were searched against the user defined database using Proteome Discoverer (Version 1.4) searching algorithm. High confidence score filter (FDR < 1%) was used to select the “hit” peptides and their corresponding MS/MS spectra were manually inspected.

### Human subjects, WES, and filtering of causative mutations

701 sporadic ALS patients were enrolled from the Department of Neurology of Peking University Third Hospital from 2007 to 2020. All ALS cases were diagnosed as possible, probable, or definite ALS according to the revised EI Escorial criteria. Clinical information, including age, sex, age of onset, site of onset, disease duration, family history and neurologic examination, were recorded. 1990 control samples for DNA analysis were obtained from the same hospital with no diagnosis of a neurological disorder. All subjects have signed the informed consent forms and this study was approved by the Ethics Committee of Peking University Third Hospital. For WES, DNA was isolated from peripheral blood using DNA Isolation Kit (Bioteke, AU1802). Genomic DNA (1 μg) were fragmented into 200–300 bp length by Covaris Acoustic System. The DNA fragments were then processed by end-repairing, A-tailing and adaptor ligation (Agilent SureSelect Human ALL Exon, V6), a 4-cycle pre-capture PCR amplification, targeted sequence capture. Captured DNA fragments were eluted and amplified by post-capture PCR. The final products were sequenced with 150–200 bp paired-end reads on Illumina HiSeq X platform according to the standard manual. The raw data produced on HiSeq X were filtered and aligned against the human reference genome (hg19) using the BWA Aligner (http://bio-bwa.sourceforge.net/v0.7.15). The single-nucleotide polymorphisms (SNPs) were called by using GATK software (Genome Analysis Toolkit, v3.6). Variants were annotated using ANNOVAR (annovar.openbioinformatics.org/en/latest/). All variants found by WES were further confirmed by Sanger sequencing. Variants were filtered for the presence of nonsynonymous heterozygous variants with a minor allele frequency (MAF) < 1% in the Exome Aggregation Consortium (ExAC) database (http://exac.broadinstitute.org/), the Exome Sequencing Project (ESP) (http://evs.gs.washington.edu), the 1000 Genomes Project (1000G) database (http://www.1000genomes.org/) and the Genome Aggregation Database (gnomAD) (http://gnomad.broadinstitute.org/). To identify the functional effect of the mutations, in silico predictive programs were performed, including Polyphen-2 (http://genetics.bwh.harvard.edu/pph2), SIFT-2 (http://sift.jcvi.org) and Mutation Taster (http://mutationtaster.org). The genomic evolutionary rate profiling scores were acquired by GERP++ program (http://mendel.stanford.edu/SidowLab/downloads/gerp/index.html).

### Quality control (QC)

After the variants were called and annotated, we applied QC steps to individuals and variants. Briefly, individual-level QC was based on common SNPs (MAF > 1%) with a genotype call rate > 95%. We excluded individuals from the association analysis who (1) were sex-discordant/ambiguous (43 individuals, 17 ALS cases and 26 controls); (2) presented a genotype call rate < 80% (0 individuals); (3) exhibited an excessive heterozygosity rate (> 3 SD from the mean; 36 individuals, 2 cases and 34 controls); (4) were shown to be ancestry outliers based on the three principal components (PCs) derived from common SNPs (0 individuals); or (5) exhibited a genetic relationship matrix value > 0.1 with another individual (32 individuals, 12 ALS patients and 20 controls). After the QC procedures, a total of 670 ALS cases and 1910 controls were retained for the analyses. We performed the same QC steps on the common capture set. After obtaining clean sets of individuals, we excluded genetic variants based on the following criteria: (1) a low genotype call rate < 99%; (2) deviation from Hardy–Weinberg equilibrium in controls (*P* < 10^–6^); (3) differential missingness between cases and controls (*P* < 10^–6^).

### Gene-based burden analysis

We assessed the evidence of an excess of rare damaging mutations in the ALS cases compared to the controls at the gene level using the sequence kernel association test (SKAT)-O implemented in the R SKAT package. We used SKAT-O because it optimally combines the burden test (which is the most powerful when a high proportion of variants in a gene are causal and exhibit the same direction of effect) with SKAT (which is best used when only a small proportion of variants in a gene are causal or if both risk and protective variants are present). Briefly, we analyzed RefSeq genes with damaging singleton sets^37^: missense variants with a MAF < 0.01% (in our dataset and East Asian populations from databases including 1000G, ESP and gnomAD non-neuro subset), and with an allele count (AC) of 1 in our data. The SKAT-O results were corrected for sex and the top ten PCs based on HapMap3 SNPs. We used the default settings in the R SKAT package, including the imputation of missing genotypes and resampling methods for computing *P* values.

## Supplementary information

Supplementary information, Fig. S1

Supplementary information, Fig. S2

Supplementary information, Fig. S3

Supplementary information, Fig. S4

Supplementary information, Fig. S5

Supplementary information, Fig. S6

Supplementary information, Fig. S7

Supplementary information, Fig. S8

Supplementary information, Fig. S9

Supplementary information, Fig. S10

Supplementary information, Fig. S11

Supplementary information, Fig. S12

Supplementary information, Fig. S13

Supplementary information, Fig. S14

Supplementary information, Fig. S15

Supplementary information, Fig. S16

Supplementary information, Fig. S17

Supplementary information, Fig. S18

Supplementary information, Fig. S19

Supplementary information, Fig. S20

Supplementary information, Fig. S21

Supplementary information, Fig. S22

Supplementary information, Fig. S23

Supplementary information, Table S1

Supplementary information, Table S2

Supplementary information, Video S1

Supplementary information, Video S2

Supplementary information, Video S3

Supplementary Video legends
