# Supplementary material for: Disruption of ER ion homeostasis maintained by an ER anion channel CLCC1 contributes to ALS-like pathologies
Source: Cell Res. 2023 May 4;33(7):497–515. doi: 10.1038/s41422-023-00798-z (PMC10313822; doi:10.1038/s41422-023-00798-z)
Supplement: Supplementary file 1 — Supplementary information, Fig. S1 [file 41422_2023_798_MOESM1_ESM.pdf]

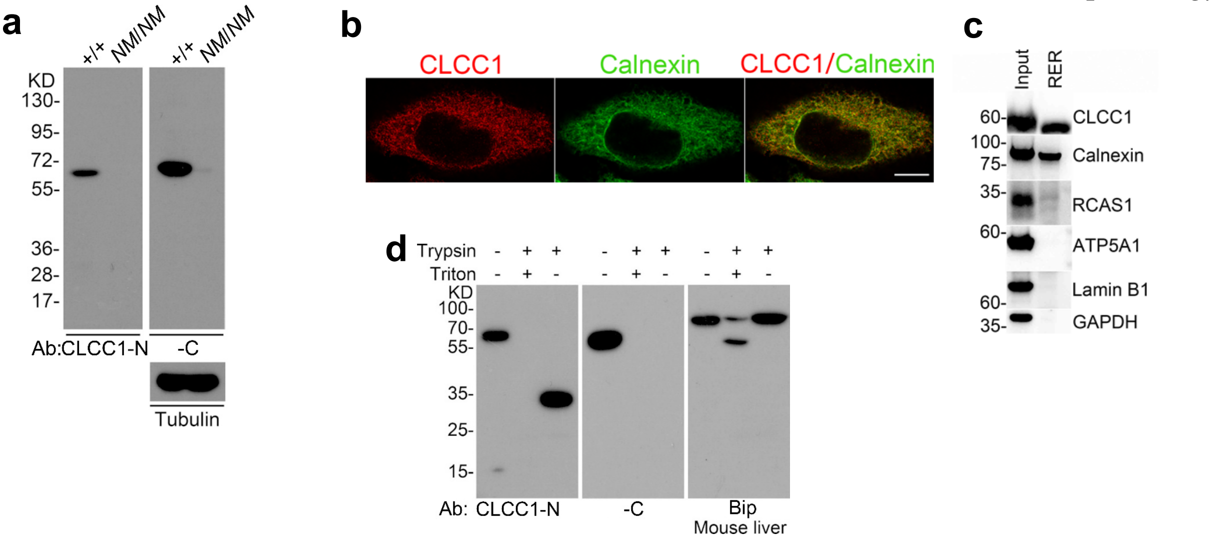

Supplementary information, Fig. S1 | CLCC1 ER localization and topology. **a**, Cerebellar lysates from wild type (+/+) and homozygous mutant *NM2453* (*NM*$^{-/-}$) mice were blotted with CLCC1 N- and C-terminal antibodies. The *NM2453* was a previously reported mutant allele that largely reduces CLCC1 expression (PMID: 25698737). Tubulin served as a loading control. **b**, Subcellular localization of CLCC1 in Hela cells was examined by the CLCC1 C-terminal antibody. Flag-tagged Calnexin, an ER resident, was detected by the Flag antibody. Scale bar, 10 μm. **c**, CLCC1 enriched in the rough ER (RER) fraction isolated by precipitation with calcium chloride. Cerebellar protein lysate of wildtype mice. Calnexin, an RER marker; RCAS1, a Golgi marker; ATP5A1, a mitochondria marker; Lamin B1, a nuclear membrane marker. **d**, Liver microsome preparations were treated with trypsin alone, or trypsin plus Triton X-100. The resulting protein lysates were then separated by SDS-PAGE and probed with the CLCC1 N- and C-terminal antibodies. An ER lumen-resident chaperone Bip was protected from trypsinization as a control.
