# Supplementary material for: Disruption of ER ion homeostasis maintained by an ER anion channel CLCC1 contributes to ALS-like pathologies
Source: Cell Res. 2023 May 4;33(7):497–515. doi: 10.1038/s41422-023-00798-z (PMC10313822; doi:10.1038/s41422-023-00798-z)
Supplement: Supplementary file 2 — Supplementary information, Fig. S2 [file 41422_2023_798_MOESM2_ESM.pdf]

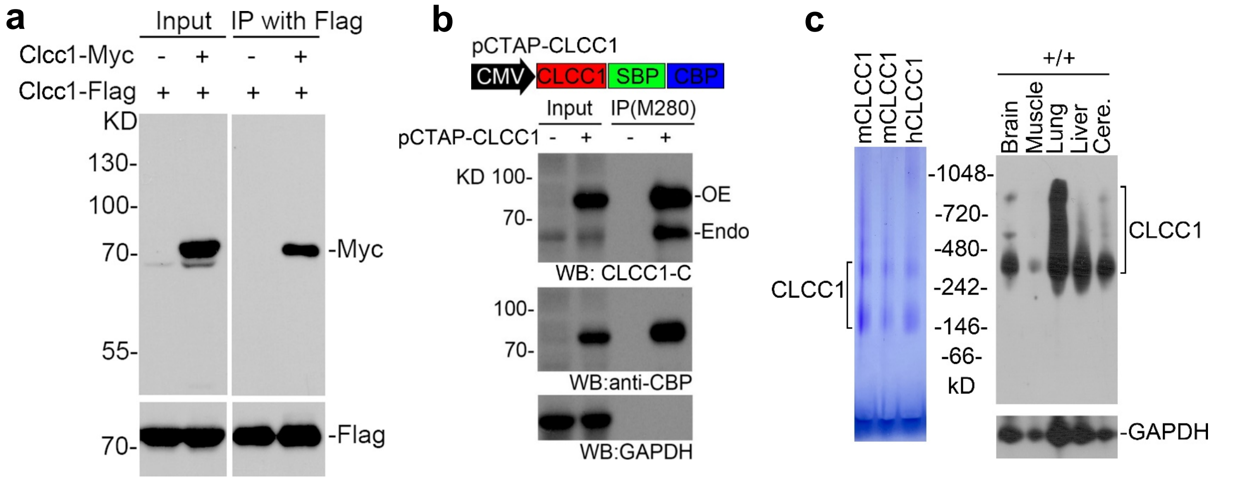

Supplementary information, Fig. S2 | Homomultimer formation of CLCC1 *in vivo* and *in vitro*. **a**, Flag-tagged mCLCC1 was expressed without or with Myc-tagged mCLCC1 in 293FT cells and the cell lysates were applied for Flag immunoprecipitation (IP) followed by western blotting for Myc. **b**, mCLCC1 tagged with SBP (streptavidin-binding peptide) and CBP (calmodulin-binding peptide) was expressed in 293FT cells. Streptavidin beads (M-280) pulled down both exogenous and endogenous CLCC1. **c**, Homomultimers of CLCC1 detected under non-reducing condition. Left, purified CLCC1; right, endogenous CLCC1. GAPDH, served as loading control.
