# Supplementary material for: Disruption of ER ion homeostasis maintained by an ER anion channel CLCC1 contributes to ALS-like pathologies
Source: Cell Res. 2023 May 4;33(7):497–515. doi: 10.1038/s41422-023-00798-z (PMC10313822; doi:10.1038/s41422-023-00798-z)
Supplement: Supplementary file 3 — Supplementary information, Fig. S3 [file 41422_2023_798_MOESM3_ESM.pdf]

a

b

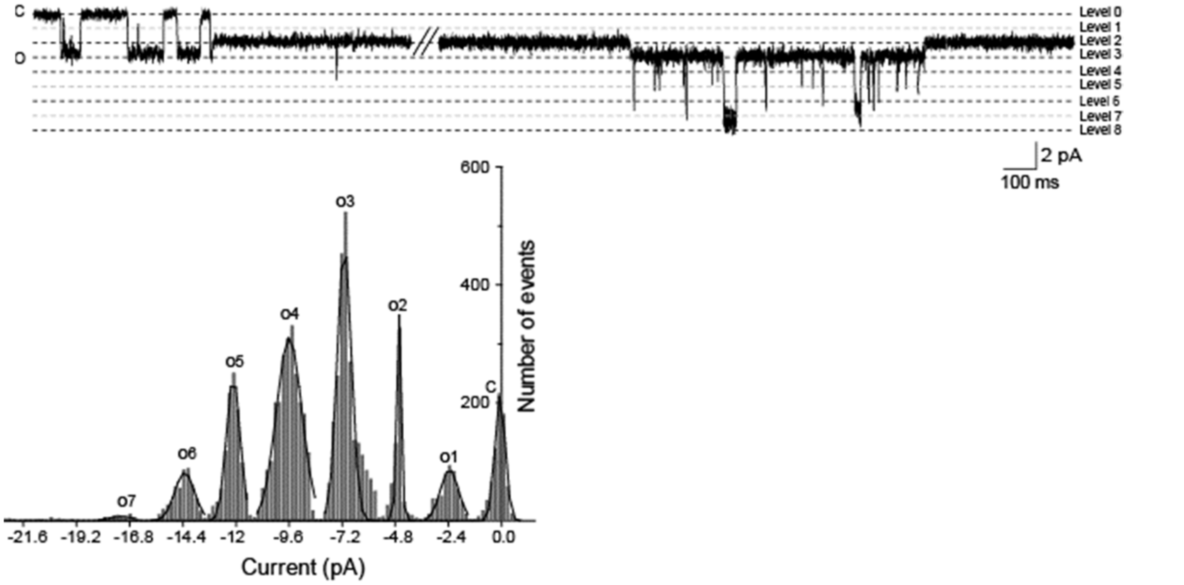

Supplementary information, Fig. S3 | Square step-like multiple-channel currents of CLCC1. **a**, Representative square step-like multiple-channel currents in asymmetric 150/15 mM KCl (In/Ex) at 0 mV. Channel opening and closing events showing eight levels of conductance. **b**, All-point amplitude histogram for the current trace (bin width = 0.05 pA/bin).
