# Supplementary material for: Disruption of ER ion homeostasis maintained by an ER anion channel CLCC1 contributes to ALS-like pathologies
Source: Cell Res. 2023 May 4;33(7):497–515. doi: 10.1038/s41422-023-00798-z (PMC10313822; doi:10.1038/s41422-023-00798-z)
Supplement: Supplementary file 4 — Supplementary information, Fig. S4 [file 41422_2023_798_MOESM4_ESM.pdf]

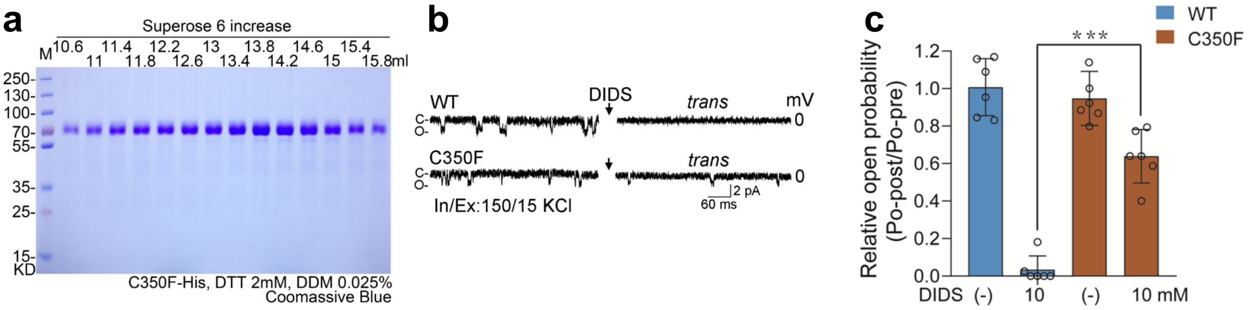

Supplementary information, Fig. S4 | C350F largely restores DIDS inhibition on CLCC1 channel activity. **a**, His-tagged C350F mutant mCLCC1 was expressed in an insect expression system and purified by Nickel column. **b**, Purified wildtype (WT) and C350F mutant mCLCC1 were incorporated into planar phospholipid bilayer and single channel recordings were performed at 0 mV in the absence and presence of 10 mM DIDS. DIDS completely inhibited the WT channel activity, which was largely restored by C350F mutant mCLCC1. **c**, Data summary of normalized open probability (*Po*). Values are presented as mean ± SD (n = 6), ***$P < 0.001$ by one-way ANOVA.
