# Supplementary material for: Disruption of ER ion homeostasis maintained by an ER anion channel CLCC1 contributes to ALS-like pathologies
Source: Cell Res. 2023 May 4;33(7):497–515. doi: 10.1038/s41422-023-00798-z (PMC10313822; doi:10.1038/s41422-023-00798-z)
Supplement: Supplementary file 5 — Supplementary information, Fig. S5 [file 41422_2023_798_MOESM5_ESM.pdf]

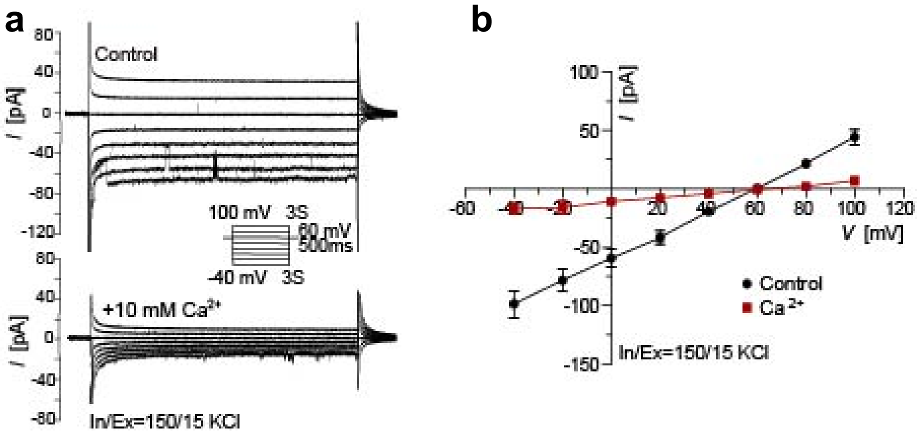

Supplementary information, Fig. S5 | Macroscopic recording for CLCC1 with or without Ca^{2+}. a and **b**, Representative macroscopic currents (**a**) and the corresponding I-V curves (**b**) recorded with or without Ca^{2+} addition (10 mM). Values are presented as mean ± SD (n = 6).
