# Supplementary material for: Disruption of ER ion homeostasis maintained by an ER anion channel CLCC1 contributes to ALS-like pathologies
Source: Cell Res. 2023 May 4;33(7):497–515. doi: 10.1038/s41422-023-00798-z (PMC10313822; doi:10.1038/s41422-023-00798-z)
Supplement: Supplementary file 6 — Supplementary information, Fig. S6 [file 41422_2023_798_MOESM6_ESM.pdf]

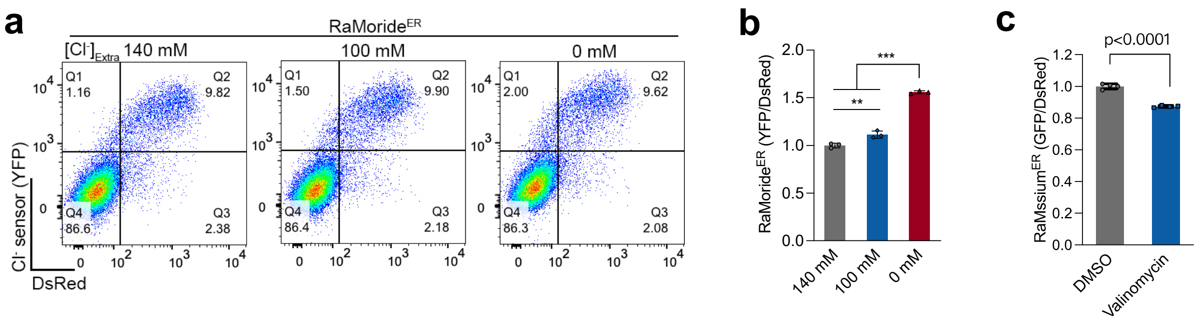

Supplementary information, Fig. S6 | Validation of RaMoride^{ER} and RaMssium^{ER} in 293FT cells. **a** and **b**, The 293FT cells expressing RaMoride^{ER} was exposed with the indicated extracellular [Cl$^-$] ([Cl$^-$]$_{Extra}$), and [Cl$^-$]$_{ER}$ was reflected by the ratio of YFP/DsRed fluorescent signals. Representative FACS (Fluorescence Activated Cell Sorting) plots (**a**) and the summary data (**b**). **c**, The 293FT cells expressing RaMssium^{ER} was resuspended in PBS with DMSO or K$^+$ ionophore (10 mM valinomycin). The [K$^+$]$_{ER}$ was reflected by the ratio of GFP/DsRed fluorescent signals. In **b** and **c**, values are presented as mean ± SD from at least three biological replicates; ***P* < 0.01, ****P* < 0.001 by one-way ANOVA or t-test.
