# Supplementary material for: Disruption of ER ion homeostasis maintained by an ER anion channel CLCC1 contributes to ALS-like pathologies
Source: Cell Res. 2023 May 4;33(7):497–515. doi: 10.1038/s41422-023-00798-z (PMC10313822; doi:10.1038/s41422-023-00798-z)
Supplement: Supplementary file 7 — Supplementary information, Fig. S7 [file 41422_2023_798_MOESM7_ESM.pdf]

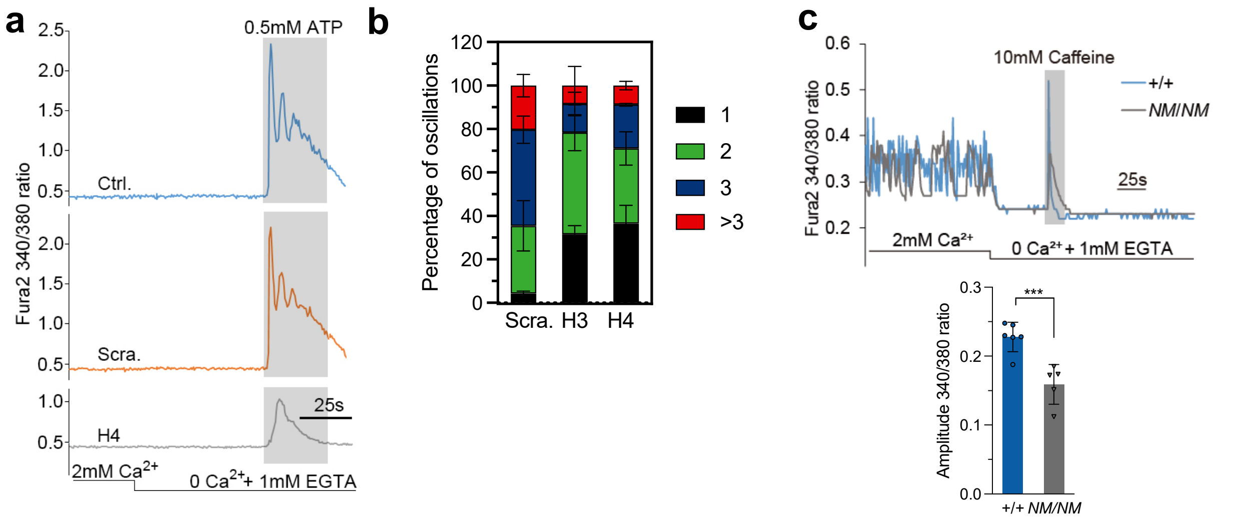

Supplementary information, Fig. S7 | Depletion of *CLCC1* impairs internal Ca^{2+} release. a, Human 293FT cells were infected with the indicated lentiviral shRNAs. The infected cells were loaded with Fura-2 and stimulated with ATP in the calcium-free medium (gray rectangles). Representative single cell Ca^{2+} traces are shown. Ctrl., MOCK control; Scra., scrambled shRNA; H4, H4 *CLCC1* shRNA. **b**, Histogram of percentage of cells showing different numbers of calcium spikes induced by ATP (at least 50 cells for each group/three independent experiments). **c**, Representative traces of intracellular Ca^{2+} changes of cardiomyocytes cultured from wildtype (+/+) and *NM2453* homozygous mutant ($NM^{-/-}$) mice, loaded with Fura-2, and stimulated with caffeine (upper). Summary data for amplitude of caffeine-induced internal Ca^{2+} release (lower). Values are presented as mean ± SD; in b, n = 3 and more than 50 cells each biological repeat; in c, n = 5, more than 15 cardiomyocytes were used per biological repeats. $^{***}P < 0.001$, by t-test.
