# Supplementary material for: Disruption of ER ion homeostasis maintained by an ER anion channel CLCC1 contributes to ALS-like pathologies
Source: Cell Res. 2023 May 4;33(7):497–515. doi: 10.1038/s41422-023-00798-z (PMC10313822; doi:10.1038/s41422-023-00798-z)
Supplement: Supplementary file 8 — Supplementary information, Fig. S8 [file 41422_2023_798_MOESM8_ESM.pdf]

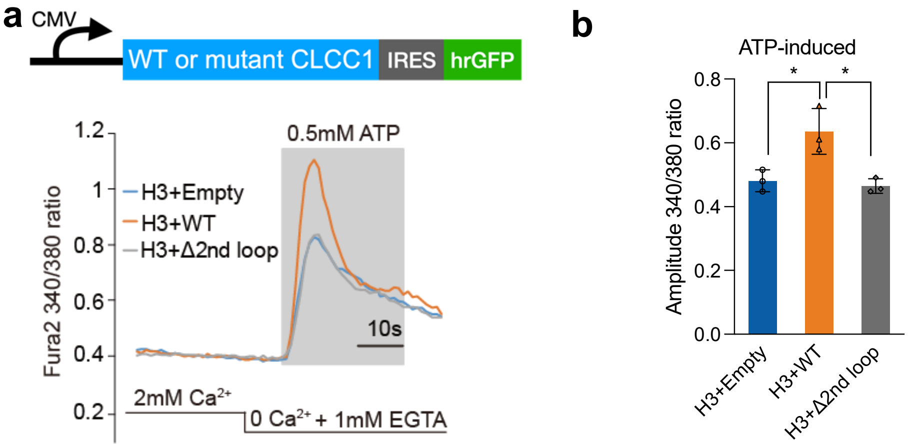

Supplementary information, Fig. S8 | The 2nd loop is responsible for the CLCC1 function. **a** and **b**, Full-length WT mCLCC1 but not the Δ2nd loop mutant CLCC1 restored the ATP-induced Ca^{2+} release damaged by H3 shRNA knockdown. The H3 knockdown stable cells were transfected with WT or Δ2nd loop expression plasmid with IRES-hrGFP. The data summary shown in (**b**). Values are presented as mean ± SD. In **b**, n =3, more than 50 cells per independent experiment. *P < 0.05, by t-test.
