# Supplementary material for: Disruption of ER ion homeostasis maintained by an ER anion channel CLCC1 contributes to ALS-like pathologies
Source: Cell Res. 2023 May 4;33(7):497–515. doi: 10.1038/s41422-023-00798-z (PMC10313822; doi:10.1038/s41422-023-00798-z)
Supplement: Supplementary file 9 — Supplementary information, Fig. S9 [file 41422_2023_798_MOESM9_ESM.pdf]

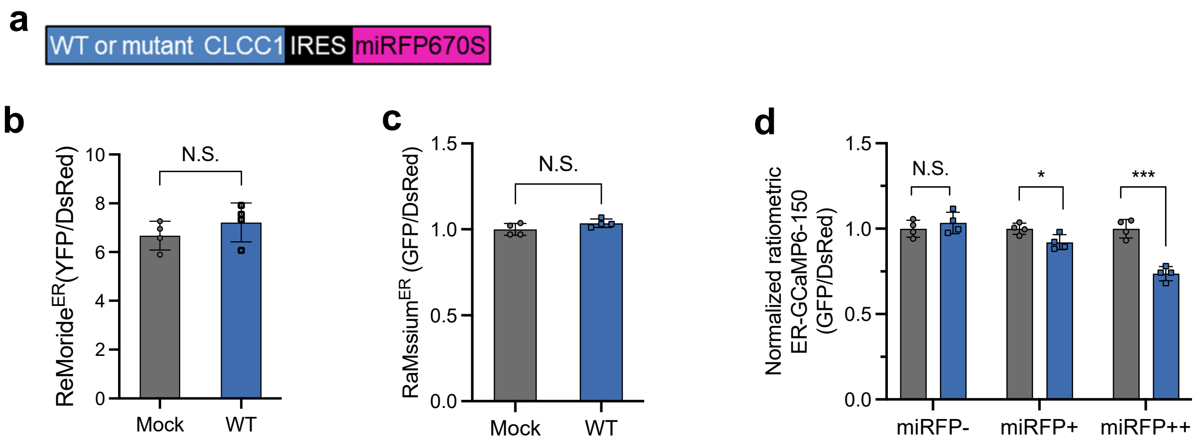

Supplementary information, Fig. S9 | Overexpression of WT CLCC1 has no effect on $[Cl^-]_{ER}$ and $[K^+]_{ER}$ but reduces $[Ca^{2+}]_{ER}$. a, The bicistronic design for transient expression of WT CLCC1. The miRFP670S was used to sort CLCC1-expressing cells. **b** and **c**, Steady state $[Cl^-]_{ER}$ (b) and $[K^+]_{ER}$ (**c**) measured by RaMoride^{ER} and RaMssium^{ER} in 293FT cells expressing WT CLCC1, respectively. **d**, Steady state $[Ca^{2+}]_{ER}$ measured by ratiometric ER-GCaMP6-150 probe in 293FT cells. The miRFP-positive subgroup cells were gated according to non-transfected cells or naïve control. In **d**, the miRFP-positive cells were divided into two groups with weak (+) and strong (++) miRFP fluorescence. Values are presented as mean ± SD. In **b-d**, n = 4. N.S., no significant difference; $^{*}P < 0.05$; $^{***}P < 0.001$; by *t*-test.
