# Supplementary material for: Disruption of ER ion homeostasis maintained by an ER anion channel CLCC1 contributes to ALS-like pathologies
Source: Cell Res. 2023 May 4;33(7):497–515. doi: 10.1038/s41422-023-00798-z (PMC10313822; doi:10.1038/s41422-023-00798-z)
Supplement: Supplementary file 10 — Supplementary information, Fig. S10 [file 41422_2023_798_MOESM10_ESM.pdf]

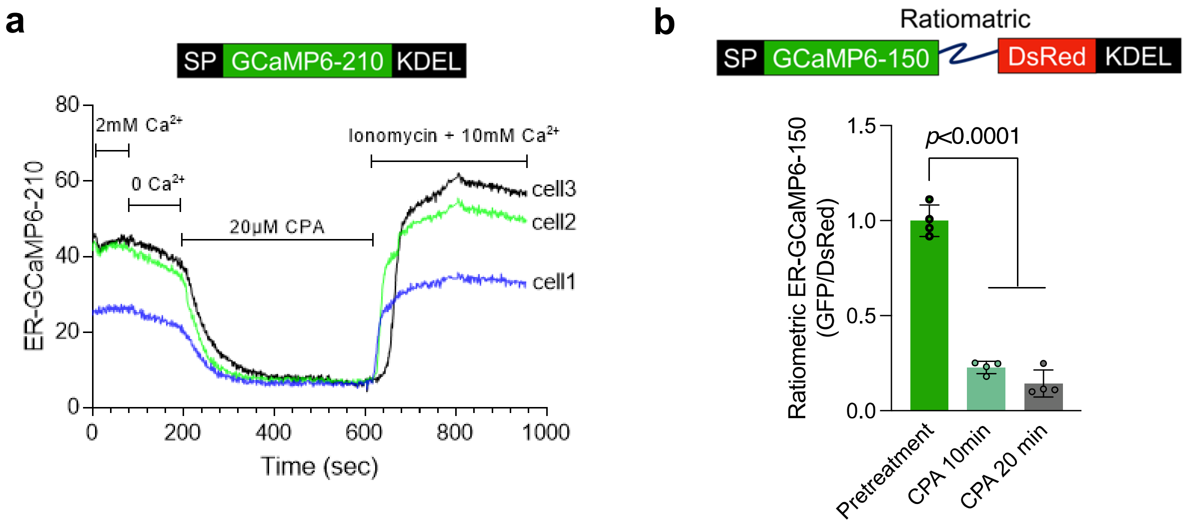

Supplementary information, Fig. S10 | Validation of an ER Ca^{2+} probe by CPA. **a**, A diagram for generation of ER-GCaMP6-210 for $[Ca^{2+}]_{ER}$ measurement (upper). The culture medium of 293FT cells expressing ER-GCaMP6-210 was sequentially switched to the indicated conditions. The fluorescent signals of ER-GCaMP6-210 dropped sharply after the application of CPA (20 μM), a Ca^{2+} pump inhibitor, which recovered after the application of 10 mM extracellular Ca^{2+} together with 10 μM ionomycin, a Ca^{2+} ionophore. Three single cell traces representing individual cells (cell1, 2, and 3) are shown (lower). **b**, A diagram for generation of ratiometric ER-GCaMP6-150 for $[Ca^{2+}]_{ER}$ measurement (upper). The 293FT cells expressing the ratiometric $[Ca^{2+}]_{ER}$ sensor were treated CPA and applied for FACS. Values are presented as mean ± SD. In **b**, n = 4, by *t*-test.
