# Supplementary material for: Disruption of ER ion homeostasis maintained by an ER anion channel CLCC1 contributes to ALS-like pathologies
Source: Cell Res. 2023 May 4;33(7):497–515. doi: 10.1038/s41422-023-00798-z (PMC10313822; doi:10.1038/s41422-023-00798-z)
Supplement: Supplementary file 11 — Supplementary information, Fig. S11 [file 41422_2023_798_MOESM11_ESM.pdf]

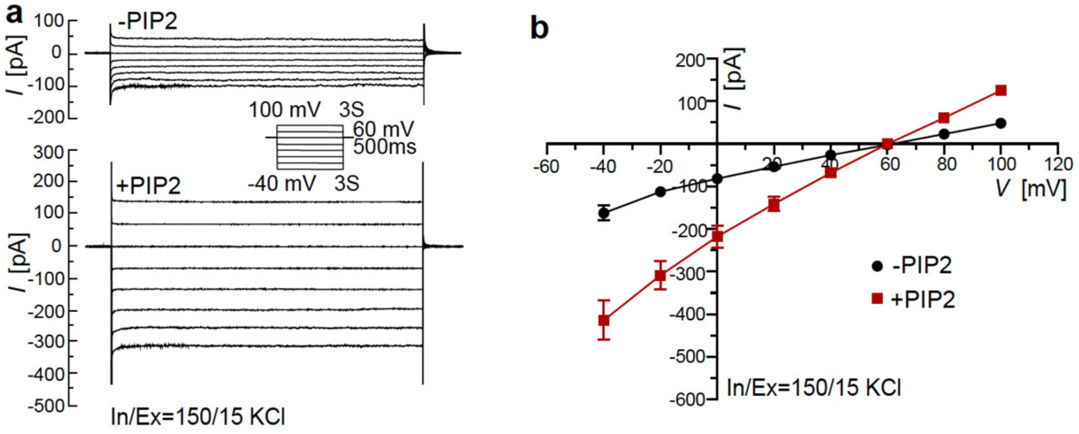

Supplementary information, Fig. S11 | Macroscopic recording for CLCC1 with or without PIP2. **a** and **b**, Representative CLCC1 macroscopic currents (**a**) and the corresponding I-V curves (**b**) recorded with or without PIP2 (2%). Values are presented as mean ± SD (n = 6).
