# Supplementary material for: Disruption of ER ion homeostasis maintained by an ER anion channel CLCC1 contributes to ALS-like pathologies
Source: Cell Res. 2023 May 4;33(7):497–515. doi: 10.1038/s41422-023-00798-z (PMC10313822; doi:10.1038/s41422-023-00798-z)
Supplement: Supplementary file 12 — Supplementary information, Fig. S12 [file 41422_2023_798_MOESM12_ESM.pdf]

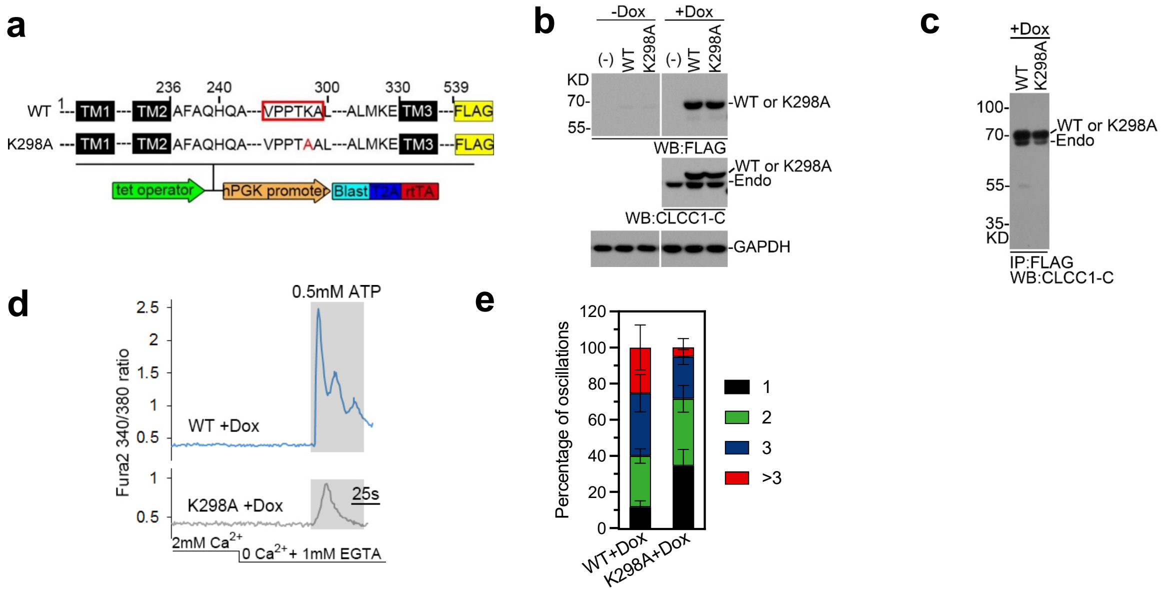

Supplementary information, Fig. S12 | K298A impairs ATP-induced Ca^{2+} oscillation. **a**, A lentiviral inducible system for expressing wildtype (WT) and K298A mutant mCLCC1 (K298A). **b**, Western blot (WB) confirmation of expression of Flag-tagged WT and K298A mutant mCLCC1 by Flag (*upper*) and CLCC1 (CLCC1-C, *middle*) antibodies in the Dox-induced cells. Endo., endogenous CLCC1. **c**, Recombinant WT and K298A mutant mCLCC1 interact with endogenous (Endo.) CLCC1. Flag-IP products were probed with the CLCC1-C antibody. **d**, Single cell calcium traces in cells with Dox induction of wildtype (WT) and mutant (K298A) mCLCC1 expression. The cells were loaded with Fura-2 and stimulated with ATP in the calcium-free culture medium (gray rectangle). **e**, Histogram of the percentage of cells showing different numbers of calcium spikes induced by ATP. At least 50 cells were analyzed for each group/three independent experiments, n=3.
