# Supplementary material for: Disruption of ER ion homeostasis maintained by an ER anion channel CLCC1 contributes to ALS-like pathologies
Source: Cell Res. 2023 May 4;33(7):497–515. doi: 10.1038/s41422-023-00798-z (PMC10313822; doi:10.1038/s41422-023-00798-z)
Supplement: Supplementary file 13 — Supplementary information, Fig. S13 [file 41422_2023_798_MOESM13_ESM.pdf]

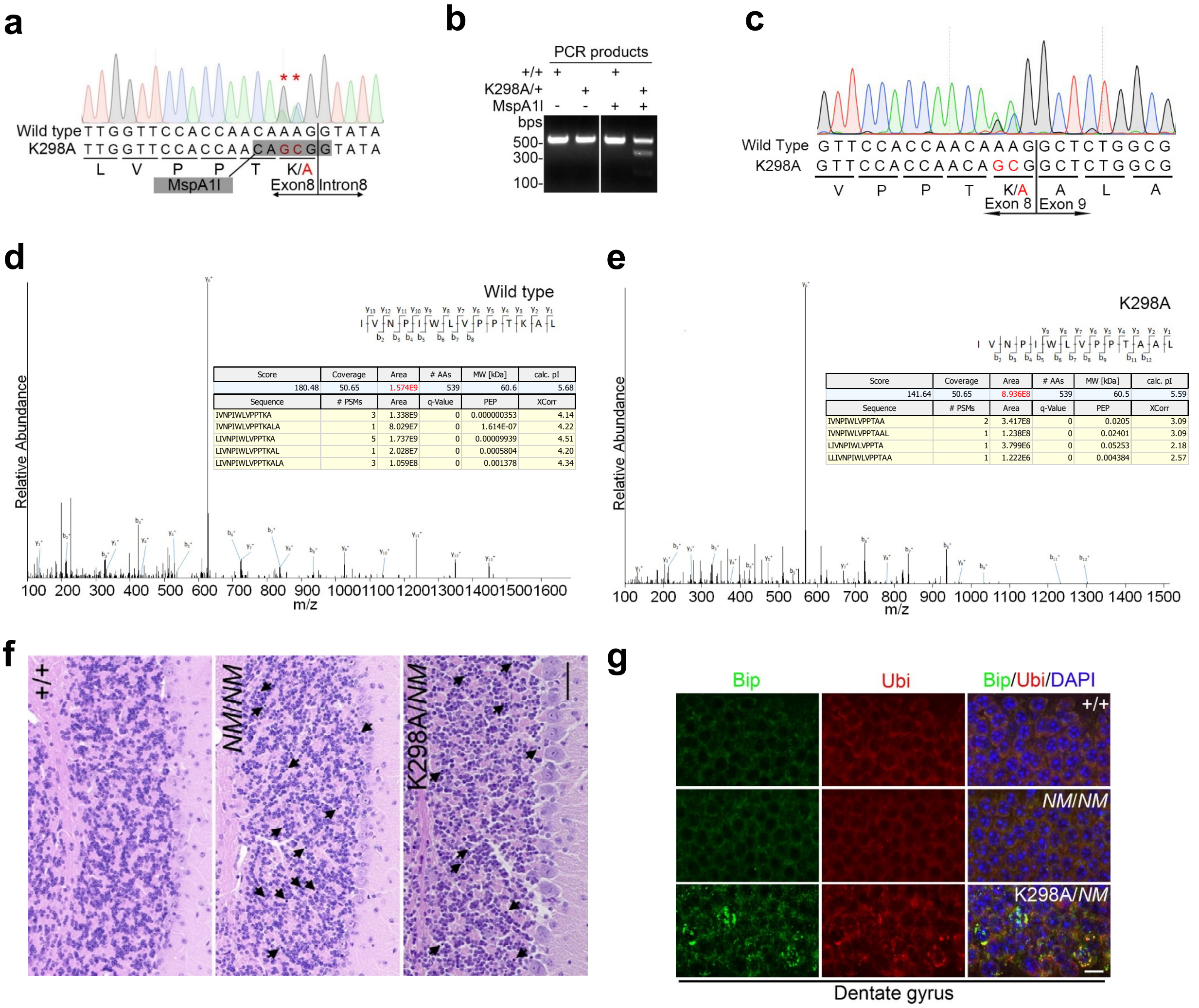

Supplementary information, Fig. S13 | K298A promotes neuron degeneration and ER stress. **a** and **b**, Generation of K298A KI mouse. Genomic DNA PCR products from the KI mutant mice were applied for Sanger sequencing (**a**) and MspA1I enzyme digestion (**b**). **c**, Cerebellar total RNA from a K298A/+ mouse was subject to RT-PCR and the PCR products were sent for Sanger sequencing. The boundary of exon 8 and 9 is marked. **d** and **e**, Brain lysates of a K298A/*NM* mouse were applied for immunoprecipitation with the CLCC1-C antibody, followed by LC-MS/MS analysis. MS/MS spectra of wildtype (**d**) and K298A CLCC1 peptides (**e**) were identified. Inserted, the quantified expression level of K298A and wild type protein by mass spectrum. **f**, Cerebellar hematoxylin and eosin (H&E) staining in mice with the indicated genotypes. The pyknotic nuclei in cerebellar granule layer were labeled with arrows. **g**, The Bip and ubiquitin (Ubi) stainings were performed in hippocampal DG areas from the indicated genotypes. In **f** and **g**, mouse age, 1.5 month. In **f**, scale bar, 50 μm; in **g**, 20 μm.
