# Supplementary material for: Disruption of ER ion homeostasis maintained by an ER anion channel CLCC1 contributes to ALS-like pathologies
Source: Cell Res. 2023 May 4;33(7):497–515. doi: 10.1038/s41422-023-00798-z (PMC10313822; doi:10.1038/s41422-023-00798-z)
Supplement: Supplementary file 14 — Supplementary information, Fig. S14 [file 41422_2023_798_MOESM14_ESM.pdf]

a

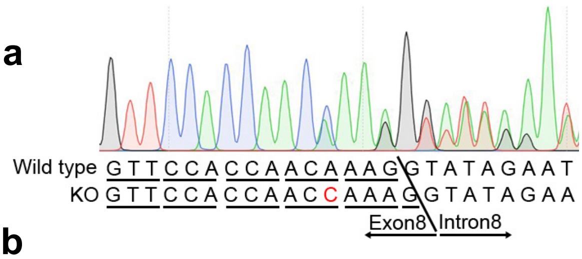

b

	+/+	KO/+	KO/KO
Expected segregation ratio	12.5	25	12.5
Observed segregation ratio	16	35	0

Note: The Chi-square value is equal to 14.59 ($p < 0.001$).

c

	+/+	K298A/+	K298A/K298A
Expected segregation ratio	13.5	27	13.5
Observed segregation ratio	20	34	0

Note: The Chi-square value is equal to 18.44 ($p < 0.001$).

Supplementary information, Fig. S14 | Abnormal Mendelian ratio in the progenies from KO/+ or K298A/+ intercross. **a**, A single nucleotide insertion generated by CRISPR/Cas9-based editing in exon 8 of *Clcc1*, which leads to out-of-frame KO. **b** and **c**, Abnormal Mendelian ratio in the progenies from KO/+ (**b**) or K298A/+ intercross (**c**).
