# Supplementary material for: Disruption of ER ion homeostasis maintained by an ER anion channel CLCC1 contributes to ALS-like pathologies
Source: Cell Res. 2023 May 4;33(7):497–515. doi: 10.1038/s41422-023-00798-z (PMC10313822; doi:10.1038/s41422-023-00798-z)
Supplement: Supplementary file 15 — Supplementary information, Fig. S15 [file 41422_2023_798_MOESM15_ESM.pdf]

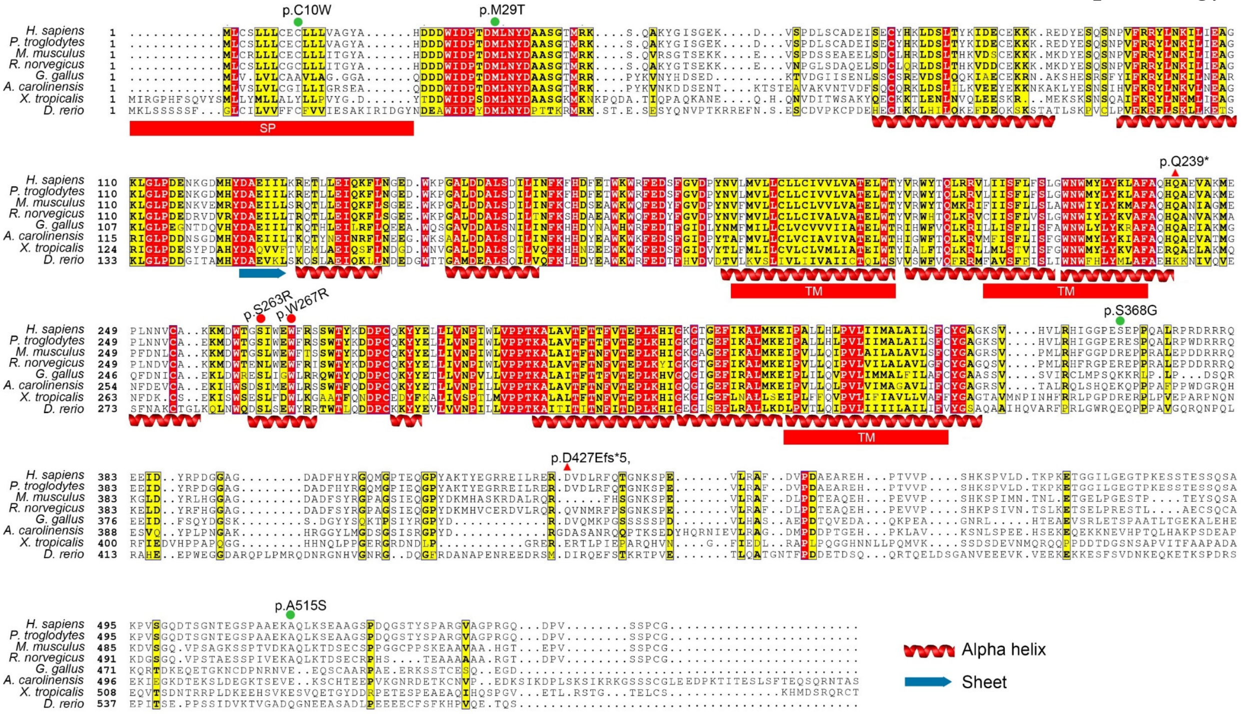

Supplementary information, Fig. S15 | The conservation and second structure prediction of CLCC1. The secondary structure, including alpha-helix and beta-sheet, was predicted by an online tool Jpred4 (PMID: 25883141). The rare genetic variances we identified in the Chinese cohort. Nonsynonymous (colored circle) and stopgain (red triangle) mutations are highlighted. Human CLCC1 sequence was used for position reference.
