# Supplementary material for: Disruption of ER ion homeostasis maintained by an ER anion channel CLCC1 contributes to ALS-like pathologies
Source: Cell Res. 2023 May 4;33(7):497–515. doi: 10.1038/s41422-023-00798-z (PMC10313822; doi:10.1038/s41422-023-00798-z)
Supplement: Supplementary file 16 — Supplementary information, Fig. S16 [file 41422_2023_798_MOESM16_ESM.pdf]

a

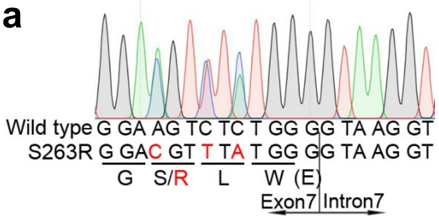

b

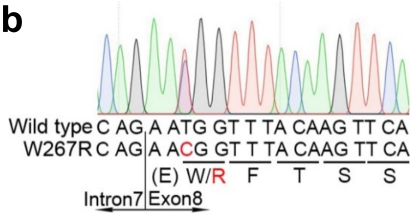

c

	+/+	S263R/+	KO/+	S263R/KO
Expected segregation ratio	13	13	13	13
Observed segregation ratio	15	25	12	0

Note: The Chi-square value was equal to 16.972 ($p < 0.001$)

d

	+/+	W267R/+	KO/+	W267R/KO
Expected segregation ratio	13.75	13.75	13.75	13.75
Observed segregation ratio	23	16	16	0

Note: The Chi-square value was equal to 16.419 ($p < 0.001$)

Supplementary information, Fig. S16 | Generation of ALS-associated mutant KI mice. a and **b**, We employed CRISPR/Cas9-mediated KI approach to generate the S263R and W267R KI mice. The genotypes of the KI mice were confirmed by genomic DNA PCR and Sanger sequencing. **c** and **d**, Abnormal Mendelian ratio in the progenies from KO/+ and S263R/+ (**c**) or W267R/+ intercross (**d**).
