# Supplementary material for: Disruption of ER ion homeostasis maintained by an ER anion channel CLCC1 contributes to ALS-like pathologies
Source: Cell Res. 2023 May 4;33(7):497–515. doi: 10.1038/s41422-023-00798-z (PMC10313822; doi:10.1038/s41422-023-00798-z)
Supplement: Supplementary file 17 — Supplementary information, Fig. S17 [file 41422_2023_798_MOESM17_ESM.pdf]

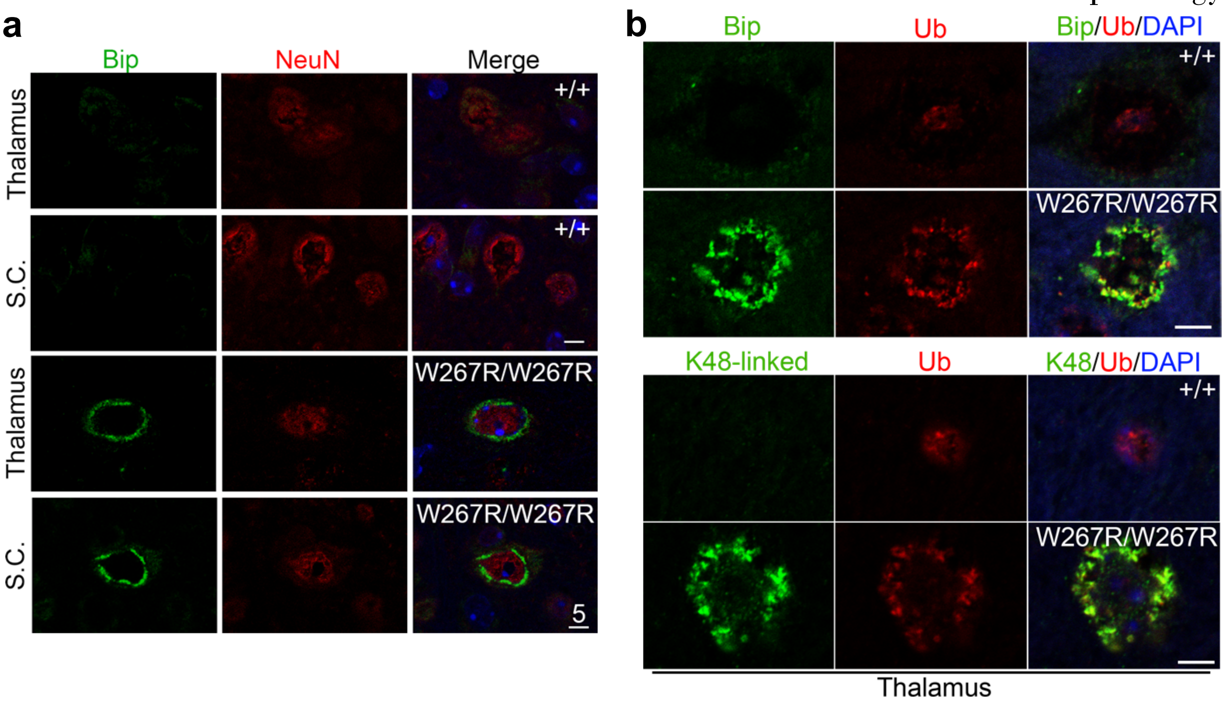

Supplementary information, Fig. S17 | Induction of neuronal ER stress in ALS-associated mutant KI mice. **a**, ER stress evidenced by Bip upregulation in thalamus and spinal cord (S.C.) neurons in W267R/W267R mice 24 hours after challenged with one dose of tunicamycin (3 mg/kg B.W.). NeuN, a neuronal maker. However, the same dose of tunicamycin did not induce the ER stress. **b**, Brain slides from the challenged animal shown in **a** were stained with the indicated antibodies. Ub, ubiquitin; K48-linked, K48-linked ubiquitination. In **a** and **b**, scale bar, 5 μm.
