# Supplementary material for: Disruption of ER ion homeostasis maintained by an ER anion channel CLCC1 contributes to ALS-like pathologies
Source: Cell Res. 2023 May 4;33(7):497–515. doi: 10.1038/s41422-023-00798-z (PMC10313822; doi:10.1038/s41422-023-00798-z)
Supplement: Supplementary file 18 — Supplementary information, Fig. S18 [file 41422_2023_798_MOESM18_ESM.pdf]

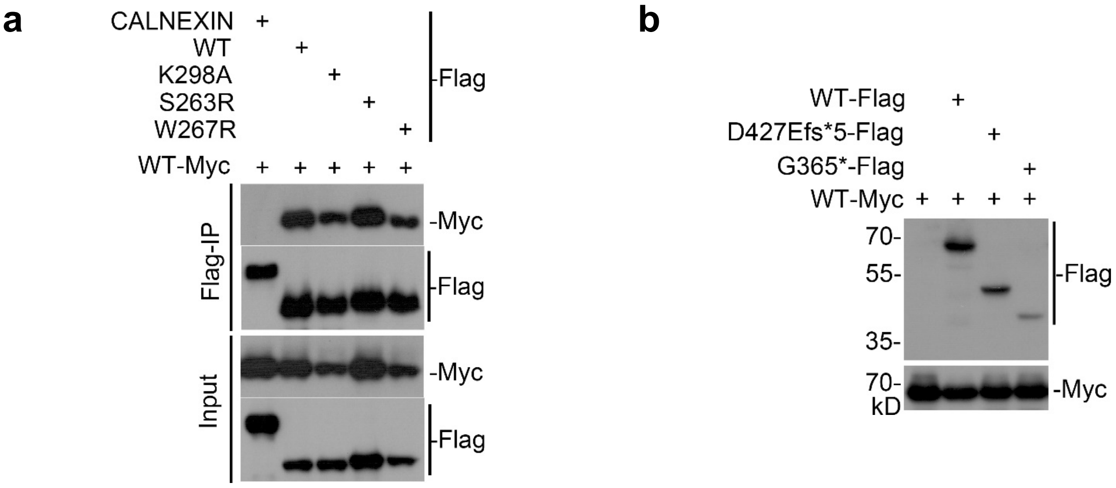

Supplementary information, Fig. S18 | The ALS-associated mutant CLCC1 interacts with wildtype in a heterologous system and C-terminus is required for full-length CLCC1 protein stability. a, Flag-tagged wildtype (WT), K298A, S263R, and W267R CLCC1 were expressed in HEK293 cells, respectively, together with myc-tagged WT CLCC1. The cell lysates were centrifuged (100,000g, 30 min) and Flag-IPed, and the resulting IP products were bolted with Flag and Myc antibodies. CALNEXIN was employed as a negative control. **b**, Flag-tagged WT and truncated CLCC1 were expressed in HEK293 cells, respectively, together with myc-tagged WT CLCC1.
