# Supplementary material for: Disruption of ER ion homeostasis maintained by an ER anion channel CLCC1 contributes to ALS-like pathologies
Source: Cell Res. 2023 May 4;33(7):497–515. doi: 10.1038/s41422-023-00798-z (PMC10313822; doi:10.1038/s41422-023-00798-z)
Supplement: Supplementary file 19 — Supplementary information, Fig. S19 [file 41422_2023_798_MOESM19_ESM.pdf]

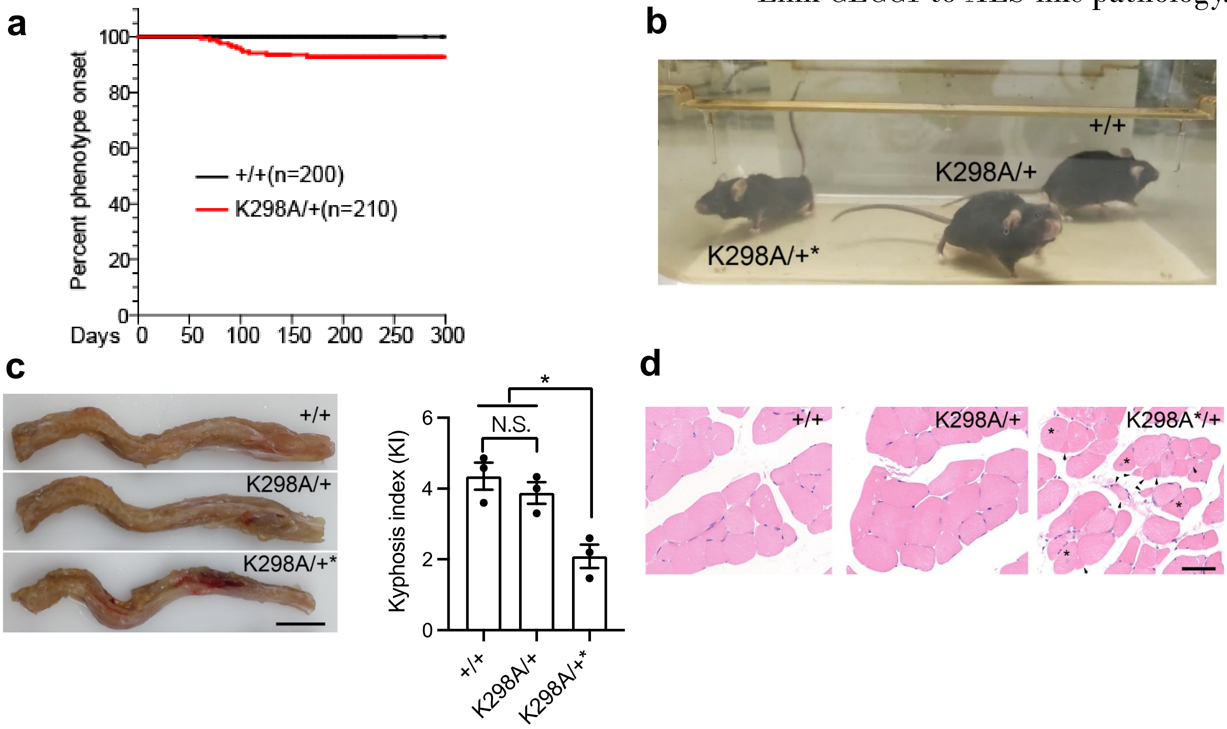

Supplementary information, Fig. S19 | Increased penetrance of K298A allele. **a**, Percent of phenotype onset in wildtype (+/+) and K298A/+ animals. The early onset phenotypes of K298A/+ animals (K298A/+*, 20/210, onset time: postnatal 90.9 $\pm$ 5.5 days) include body weight loss, hindlimb weakness, trunk shaking, tail flagging, abnormal gaits, and ataxia (also see Extended Data Movie 3). **b**, A K298A/+ mutant mouse displaying early onset phenotype (K298A/+*) together with a wildtype and a normal K298A/+ mouse. **c**, Curved spine (kyphosis) shown in K298A/+* but not in wildtype and K298A/+ mice. Kyphosis index (KI) was employed to quantitatively reflect the kyphosis (PMID: 15234960). **d**, Hematoxylin-and eosin-stained cross sections of gastrocnemius muscles from +/+, K298A/+, and K298A/+* mice. Arrowheads and asterisks indicate atrophied muscle fibers and fibers with central nuclei, respectively. In **c** and **d**, mouse, male, 10 months of age. Values are presented as mean $\pm$ SD from three independent animals; * $p < 0.05$, by one-way ANOVA.
