# Supplementary material for: Disruption of ER ion homeostasis maintained by an ER anion channel CLCC1 contributes to ALS-like pathologies
Source: Cell Res. 2023 May 4;33(7):497–515. doi: 10.1038/s41422-023-00798-z (PMC10313822; doi:10.1038/s41422-023-00798-z)
Supplement: Supplementary file 20 — Supplementary information, Fig. S20 [file 41422_2023_798_MOESM20_ESM.pdf]

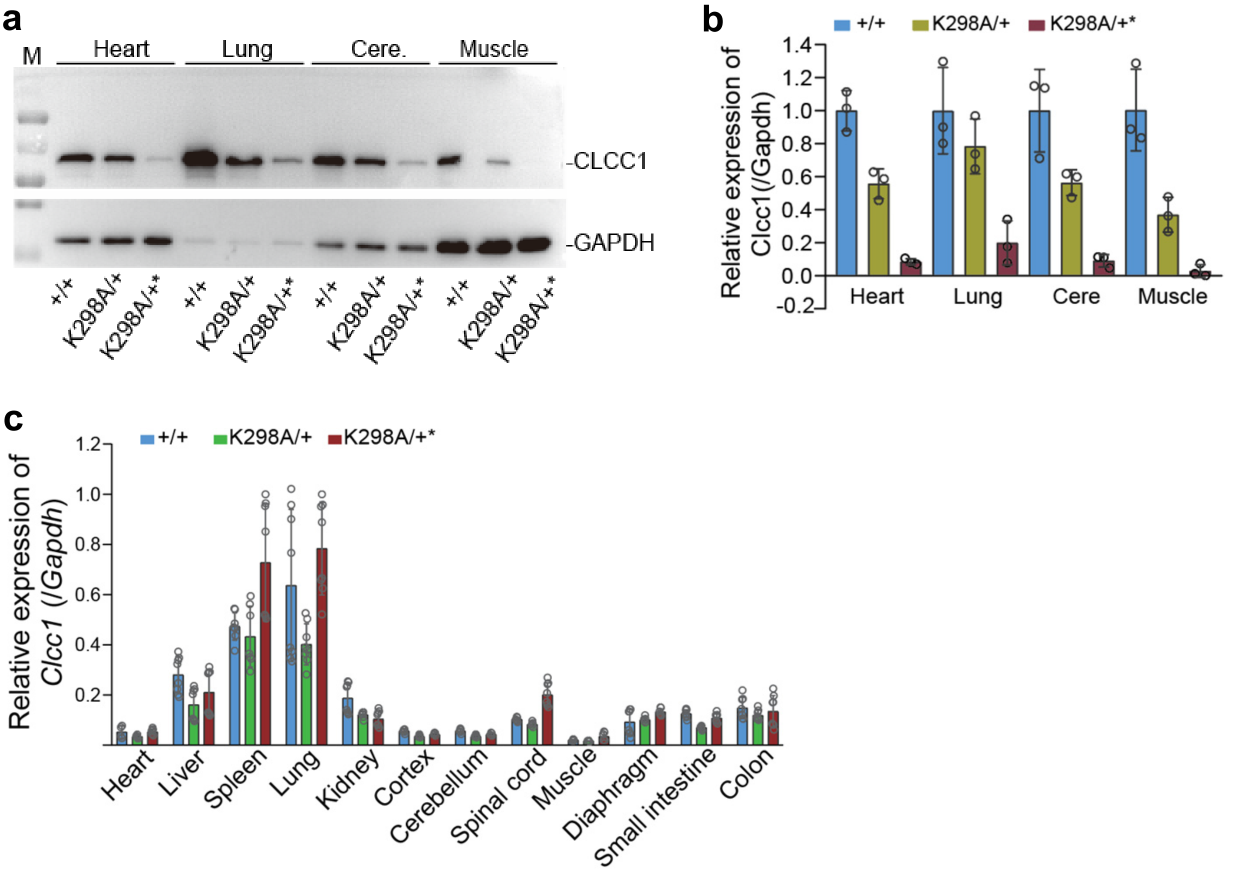

Supplementary information, Fig. S20 | Protein and mRNA expression of *Clcc1* in K298A/+ mice with/without early onset phenotypes. **a** and **b**, Expression levels of CLCC1 in various tissues in wildtype, K298A/+, and K298A/+* mice. Summary data in **b**. Expression levels of CLCC1 were normalized to GAPDH. **c**, *Clcc1* mRNA levels were detected by real-time PCR in the various tissues with indicated genotypes and phenotypes. Mouse, male, 10 months of age. Values are presented as mean ± SD from three independent experiments; * $p < 0.05$, *** $p < 0.001$, by one-way ANOVA.
