# Supplementary material for: Disruption of ER ion homeostasis maintained by an ER anion channel CLCC1 contributes to ALS-like pathologies
Source: Cell Res. 2023 May 4;33(7):497–515. doi: 10.1038/s41422-023-00798-z (PMC10313822; doi:10.1038/s41422-023-00798-z)
Supplement: Supplementary file 21 — Supplementary information, Fig. S21 [file 41422_2023_798_MOESM21_ESM.pdf]

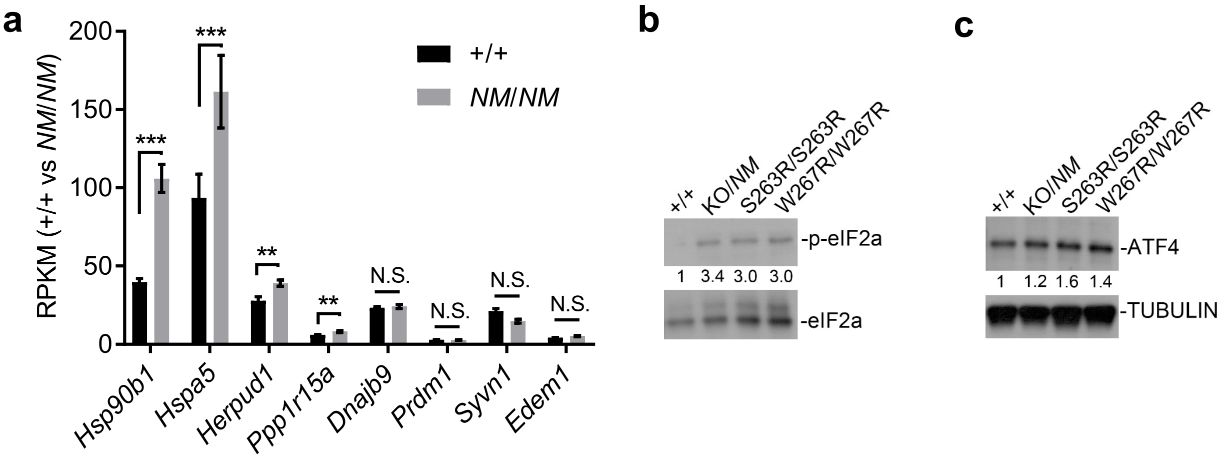

Supplementary information, Fig. S21 | Primary activations of PERK-eIF2a-ATF4 and ATF6 pathways but not IRE1a-XBP1 pathway upon dysfunction/depletion of CLCC1. a, Expression levels (RPKM) of ATF6 target genes (*Hsp90b1*, *Hspa5*, *Herpud1*, and *Ppp1r15a*) and XBP1 target genes (*Dnajb9*, *Prdm1*, *Syvn1*, and *Edem1*) in the *NM*/*NM* and +/+ cerebella. RNA-seq data generated from +/+ and *NM*/*NM* cerebella. Animals, P30 males (n=3). **b** and **c**, Expression levels of phosphorylated-eIF2α (p-eIF2α) and ATF4 in the cerebella with the indicated genotypes measured by western blot. Expression levels of eIF2α and TUBULIN were employed as loading controls for p-eIF2α and ATF4, respectively. Animals, P30 males. Normalized band densities were labeled. In **a**, values are presented as mean ± SD; ** $p < 0.01$, *** $p < 0.001$, N.S., no significant difference, by t-test.
