# Supplementary material for: Disruption of ER ion homeostasis maintained by an ER anion channel CLCC1 contributes to ALS-like pathologies
Source: Cell Res. 2023 May 4;33(7):497–515. doi: 10.1038/s41422-023-00798-z (PMC10313822; doi:10.1038/s41422-023-00798-z)
Supplement: Supplementary file 22 — Supplementary information, Fig. S22 [file 41422_2023_798_MOESM22_ESM.pdf]

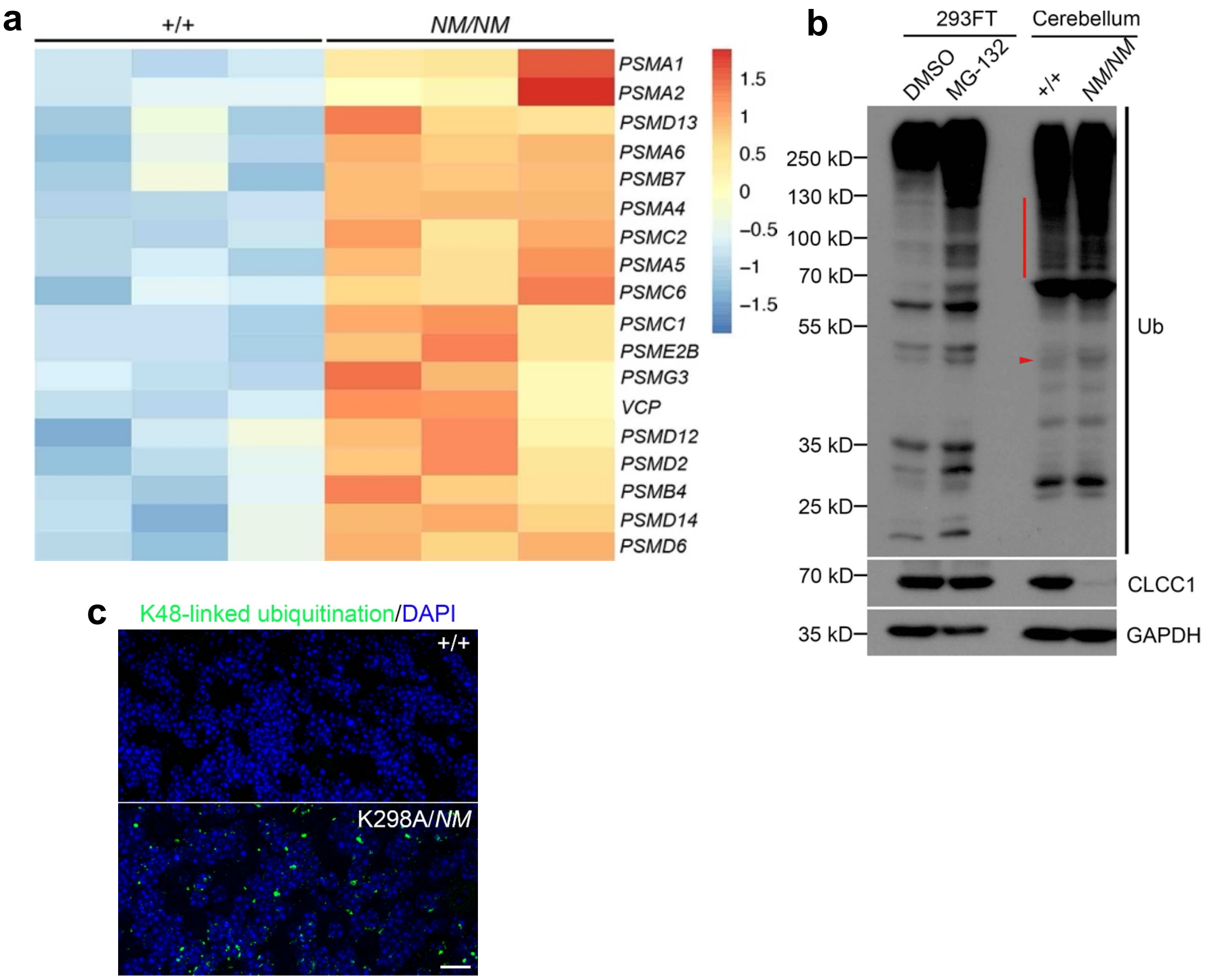

Supplementary information, Fig. S22 | Dysfunction of CLCC1 leads to activation of proteasome-mediated protein degradation. **a**, Upregulation of genes involved in proteasome function. Cerebella of wild type (+/+) and *NM*/*NM* animals (P30 male) were applied for RNA-seq. Z-score was used to normalize the expression in each row. **b**, Global ubiquitination detected in the *NM*/*NM* cerebellum. Cerebella of wildtype (+/+) and *NM*/*NM* animals (P30 male) were applied for western blot. HEK293FT cells were treated with MG-132 for 60 minutes as a global ubiquitination positive control. GAPDH served as loading controls. The global ubiquitination in the *NM*/*NM* cerebellum is labeled with red line or arrow. **c**, K48-linkage specific poly-ubiquitination detected in the K298A/NM cerebellum. Cerebella of wild type (+/+) and K298A/*NM* animals (P30 male) were applied for the immunostaining. In **c**, scale bar, 20 μm.
