# Supplementary material for: Disruption of ER ion homeostasis maintained by an ER anion channel CLCC1 contributes to ALS-like pathologies
Source: Cell Res. 2023 May 4;33(7):497–515. doi: 10.1038/s41422-023-00798-z (PMC10313822; doi:10.1038/s41422-023-00798-z)
Supplement: Supplementary file 23 — Supplementary information, Fig. S23 [file 41422_2023_798_MOESM23_ESM.pdf]

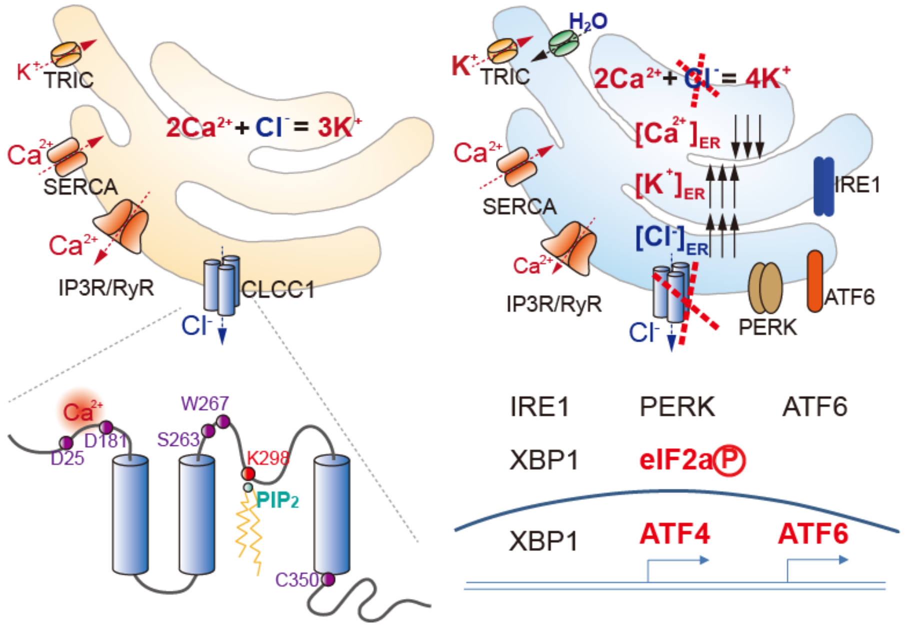

Supplementary information, Fig. S23 | Our working model. The working model for CLCC1 in regulations of ER ion homeostasis, ER morphology, and ER stress. Top left, ER Ca^{2+} release through the release channels (IP_3Rs and/or RyRs) positively charges the ER membrane, which in turn decreases the driving force for continued Ca^{2+} release. Cl^- efflux through CLCC1 and K^+ influx via TRIC channels or IP3Rs/RyRs work together to compensate both ER membrane potential and luminal osmolarity changes during the release. CLCC1 functions as homomultimers. High [Ca^{2+}] in the ER lumen (represented as light brown) inhibits CLCC1 channel activity. Topologically, CLCC1 N-terminus and the 2nd loop face ER lumen and its C-terminus is at cytoplasmic side (lower enlarged). Key residues of CLCC1 for its anion channel activity are labeled. D25/D181 are the key residues for Ca^{2+}-binding and Ca^{2+}-dependent inhibition on the channel activity. K298 in the second loop for PIP2-facilitation of the channel activity. Two ALS-associated mutations (S263R and W267R) are located in the same loop. MTSET and DIDS work on C350 to affect CLCC1 channel activity, suggesting that C350 is close to the conduction pathway. Top right, upon dysfunction of CLCC1, $[Cl^-]_{ER}$ is increased and the counter movement of Cl^- through CLCC1 during the store Ca^{2+} release is impaired, which in turn reduces the driving force of Ca^{2+} release. Although pure K^+ influx can also partially compensate for the loss of ER membrane potential, it increase luminal osmolarity. High $[Cl^-]_{ER}$ and $[K^+]_{ER}$ enlarge ER volume due to the uptake of H_2O through osmosis and decreases [Ca^{2+}]ER, high level of which is crucial for correct ER protein folding. Dysfunction of CLCC1 leads to ER unfolded protein response mainly through PERK-eIF2α-ATF4 and ATF6 pathways. SERCA, sarco/endoplasmic reticulum Ca^{2+}-ATPase responsible for transferring Ca^{2+} from cytosol to ER lumen.
