# Supplementary material for: Disruption of ER ion homeostasis maintained by an ER anion channel CLCC1 contributes to ALS-like pathologies
Source: Cell Res. 2023 May 4;33(7):497–515. doi: 10.1038/s41422-023-00798-z (PMC10313822; doi:10.1038/s41422-023-00798-z)
Supplement: Supplementary file 24 — Supplementary information, Table S1 [file 41422_2023_798_MOESM24_ESM.pdf]

**Supplementary information, Table S1 | Rare genetic variances found in ALS patients in a Chinese cohort.**

| No. | rsID | Chr1 | Mutation | Category | Patient | Control | ExAC | GnomAD | TOPMED | 1000G |
|---|---|---|---|---|---|---|---|---|---|---|
| 1 | N.A. | 109484036 | c.787A>C, p.Ser263Arg | Nonsynonymous | 2 | 0 | N.A. | N.A. | N.A. | N.A. |
| 2 | N.A. | 109482762 | c.799T>C, p.Trp267Arg | Nonsynonymous | 1 | 0 | N.A. | N.A. | N.A. | N.A. |
| 3 | N.A. | 109479980 | c.1102A>G, p.Ser368Gly | Nonsynonymous | 1 | 0 | N.A. | N.A. | N.A. | N.A. |
| 4 | N.A | 109492974 | c.86T>C p. Met29Thr | Nonsynonymous | 1 | 0 | N.A | N.A | N.A | N.A |
| 5 | rs750385149 | 109479801 | c.1280_1281insGA, p.Asp427Glufs*5 | Stop gain | 1 | 0 | N.A. | N.A. | N.A. | N.A. |
| 6 | rs202199249 | 109477405 | c.1543G>T, p.Ala515Ser | Nonsynonymous | 1 | 0 | N.A. | 4.06E-06 | 7.96E-06 | 2.00E-04 |
| 7 | rs770795683 | 109484108 | c.715C>T, p.Gln239* | Stop gain | 1 | 0 | 8.25E-06 | 4.06E-06 | 3.19E-05 | N.A. |
| 8 | rs372449194 | 109493031 | c.29G>A, p.Cys10Tyr | Nonsynonymous | 1 | 0 | 1.66E-05 | 1.63E-05 | 7.96E-06 | N.A. |

Note: The genetic variances were detected in a Chinese cohort which contains 670 ALS sporadic patients and 1910 controls without neurological diseases. ExAC, The Exome Aggregation Consortium; GnomAD, The Genome Aggregation Database; TOPMed, The Trans-Omics for Precision Medicine program; 1000G, The 1000 Genomes Project. The chromosome position is based on human reference sequence (GRCh37, 2009).
