# Supplementary material for: Disruption of ER ion homeostasis maintained by an ER anion channel CLCC1 contributes to ALS-like pathologies
Source: Cell Res. 2023 May 4;33(7):497–515. doi: 10.1038/s41422-023-00798-z (PMC10313822; doi:10.1038/s41422-023-00798-z)
Supplement: Supplementary file 25 — Supplementary information, Table S2 [file 41422_2023_798_MOESM25_ESM.pdf]

Supplementary information, Table S2 | Clinical records of the patients with CLCC1 mutations.

Mutation	Age of 1st visit	Sex	Described onset age	Delayed time of diagnosis (mos.)	Current status	Survival time (mos.)	Onset position	Description of Onset	ALS or FTD/ALS
p.Q239*	35	M	34	17	Dead	78	Cervical	Right hand weakness	ALS
p.S263R	40	M	39	6	Dead	53	Lumbar	Left lower limb weakness	ALS
p.S263R	46	M	45	10	Dead	38	Lumbar	Right lower limb weakness	ALS
p.W267R	76	F	74	31	Dead	61	Lumbar	Right lower limb weakness	ALS
p.D427Efs*5	51	M	50	8	Dead	44	Cervical	Left hand weakness	ALS
p.C10W	38	F	37	10	N.A.	-	Cervical	Right hand weakness	ALS
p.M29T	27	F	17	6	Survival	122	Lumbar	Lower limb weakness	ALS
p.S368G	64	M	61	43	Dead	94	Cervical	Right hand weakness	ALS
p.A515S	22	M	17	6	Dead	135	Lumbar	Left lower limb weakness	ALS
