# Supplementary material for: Disruption of ER ion homeostasis maintained by an ER anion channel CLCC1 contributes to ALS-like pathologies
Source: Cell Res. 2023 May 4;33(7):497–515. doi: 10.1038/s41422-023-00798-z (PMC10313822; doi:10.1038/s41422-023-00798-z)
Supplement: Supplementary file 29 — Supplementary Video legends [file 41422_2023_798_MOESM29_ESM.pdf]

Supplementary information, Video S1 Early onset behavior phenotypes in the K298A/*NM* mouse. Mice with indicated genotypes were videotaped in side view. K298A/*NM* mouse showed severe body weight loss, hind leg weakness, trunk shaking, tail flagging, abnormal gaits, and ataxia, which were not shown in aged-matched WT and *NM*/*NM* mice. Obvious phenotypes appeared in the *NM*/*NM* mice at the age of 1.5 year (PMID: 25698737).

Supplementary information, Video S2 Early onset phenotypes shown in the KO/*NM* mouse. KO/*NM* mouse (2.5-month old) was shown in left corner at the binging of the videotape. K298A/*NM* mouse (4.5-month old) was then put into the field, which carried less motor phenotypes than the KO/*NM* mouse.

Supplementary information, Video S3 Increased penetrance of K298A allele. A K298A/+ mutant mouse carrying early onset phenotype (K298A/+*), which is similar to that of K298A/*NM* (**Supplementary information, Video S1**), together with a wild-type and a normal K298A/+ mouse. Mouse, male, 10 months of age.
